# Dissociable Effects of Serotonin and Dopamine on the Valuation of Harm in Moral Decision Making

**DOI:** 10.1016/j.cub.2015.05.021

**Published:** 2015-07-20

**Authors:** Molly J. Crockett, Jenifer Z. Siegel, Zeb Kurth-Nelson, Olga T. Ousdal, Giles Story, Carolyn Frieband, Johanna M. Grosse-Rueskamp, Peter Dayan, Raymond J. Dolan

**Affiliations:** 1 Department of Experimental Psychology, University of Oxford, 9 South Parks Road, Oxford OX1 3UD, UK; 2 Wellcome Trust Centre for Neuroimaging, University College London, 12 Queen Square, London WC1N 3BG, UK; 3 Max Planck-UCL Centre for Computational Psychiatry and Ageing, University College London, 12 Queen Square, London WC1N 3BG, UK; 4 Department of Radiology, Haukeland University Hospital, Jonas Lies vei 65, 5021 Bergen, Norway; 5 Gatsby Computational Neuroscience Unit, University College London, Alexandra House, 17 Queen Square, London WC1N 3AR, UK

## Abstract

An aversion to harming others is a core component of human morality and is disturbed in antisocial behavior [1–4]. Deficient harm aversion may underlie instrumental and reactive aggression, which both feature in psychopathy [5]. Past work has highlighted monoaminergic influences on aggression [6–11], but a mechanistic account of how monoamines regulate antisocial motives remains elusive. We previously observed that most people show a greater aversion to inflicting pain on others than themselves [12]. Here, we investigated whether this hyperaltruistic disposition is susceptible to monoaminergic control. We observed dissociable effects of the serotonin reuptake inhibitor citalopram and the dopamine precursor levodopa on decisions to inflict pain on oneself and others for financial gain. Computational models of choice behavior showed that citalopram increased harm aversion for both self and others, while levodopa reduced hyperaltruism. The effects of citalopram were stronger than those of levodopa. Crucially, neither drug influenced the physical perception of pain or other components of choice such as motor impulsivity or loss aversion [13, 14], suggesting a direct and specific influence of serotonin and dopamine on the valuation of harm. We also found evidence for dose dependency of these effects. Finally, the drugs had dissociable effects on response times, with citalopram enhancing behavioral inhibition and levodopa reducing slowing related to being responsible for another’s fate. These distinct roles of serotonin and dopamine in modulating moral behavior have implications for potential treatments of social dysfunction that is a common feature as well as a risk factor for many psychiatric disorders.

## Highlights

• Serotonin and dopamine had distinct effects on decisions to harm self versus others
• Computational models revealed a hyperaltruistic preference to harm self over others
• Pharmacological enhancement of serotonin increased harm aversion for self and others
• Pharmacological enhancement of dopamine reduced hyperaltruism

## Teaser

Crockett et al. pharmacologically manipulated serotonin and dopamine levels in healthy participants to investigate the neuromodulation of decisions to harm self and others. Enhancing serotonin function increased harm aversion for self and others, while enhancing dopamine function reduced a hyperaltruistic preference to harm oneself over others.

## Results

Many aspects of the way the human brain carries out the computations essential for healthy social interactions remain unclear. Overlapping neural representations of the value of one’s own and others’ outcomes are a central component of empathy [15] and prosocial behavior [16], suggesting that attention be paid to neural systems involved in valuation. Central among these are the neuromodulators serotonin and dopamine. Indeed, many psychiatric disorders associated with monoaminergic abnormalities feature social dysfunction [1, 11, 17], and interactions between serotonin and dopamine have been implicated in impulsive aggression [18] and psychopathy [19]. However, previous studies have primarily examined how these neuromodulators influence the valuation of outcomes for oneself [20, 21]. How these systems influence the valuation of others’ outcomes—particularly harmful ones—is not well understood. A monoaminergic influence on the valuation of harm to others may explain the link between monoamines and aggression that has been observed across species [6–11]. Prior work suggests an influence of serotonin on harm aversion. Deciding whether to harm others engages brain regions densely innervated by serotonin [8, 22, 23], and manipulating serotonin function influences the expression of harm aversion in hypothetical moral judgments [24], though these are not necessarily predictive of real moral decisions [3]. A role for dopamine in harm aversion is less clear. Although biomarkers of hyperactive mesolimbic dopamine function in humans correlate with trait aggression [10] and impulsive-antisocial psychopathic traits [11, 19], direct evidence supporting a causal influence of dopamine on human antisocial behavior is sparse. Previous studies have shown dopaminergic effects on economic decisions [25, 26], but existing economic models are poor predictors of moral decisions concerning harm to others [12].

We recently developed a method for quantifying how people value the pain of others relative to their own pain and observed that most people were “hyperaltruistic,” requiring more financial compensation to inflict pain on a stranger than themselves [12]. Here, we investigated how serotonin and dopamine modulate hyperaltruism and the valuation of harm to self and others. In light of past studies suggesting serotonin enhances non-social aversive processing [27–29], we predicted that enhancing serotonin function would increase harm aversion for both oneself and others. Meanwhile, given previous work showing positive correlations between high mesolimbic dopaminergic tone and antisocial behavior [10, 11], we predicted that enhancing dopamine function would reduce a hyperaltruistic tendency to prefer harming oneself over harming others.

We tested these hypotheses using double-blind pharmacological manipulations of serotonin and dopamine in subjects performing a moral decision task that allowed us to quantify harm aversion for self and others [12]. In study 1, 89 healthy volunteers received either placebo or 30 mg of the selective serotonin reuptake inhibitor citalopram, which enhances serotonin neurotransmission by blocking its reuptake and prolonging its actions in the synapse [30]. In study 2, 86 healthy volunteers received either placebo or 150 mg of the dopamine precursor levodopa, which elevates central dopamine levels [30]. The decision task was timed to coincide with peak drug absorption.

**Figure 1:**
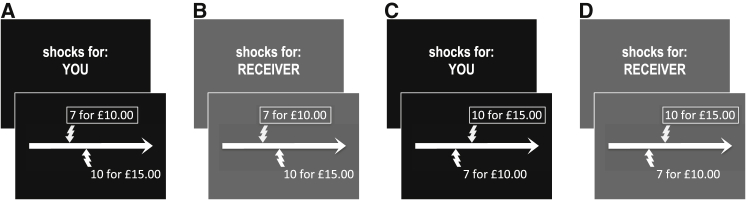
Experimental Design In each trial, deciders chose between less money and fewer shocks versus more money and more shocks. (A–D) The money was always for the decider, but in half the trials, the shocks were allocated to the decider (A and C), and, in the other half, the shocks were allocated to the receiver (B and D). In all trials, if the decider failed to press a key within 6 s, the highlighted default (top) option was registered; if the decider pressed the key, the alternative (bottom) option was highlighted and registered instead. In half the trials, the alternative option contained more money and shocks than the default (A and B), so action resulted in greater harm and profit. In the other half, the alternative option contained less money and shocks than the default (C and D), so inaction resulted in greater harm and profit.

We first used a standard procedure to determine each subject’s pain threshold for an electrical shock stimulus delivered to the left wrist [31]. This thresholding enabled us to create a bespoke shock stimulus for each subject that was mildly painful, but not intolerable, and matched in subjective intensity for all subjects. Immediately after the thresholding, subjects played the role of “decider” in a decision task (Figure 1) where they made 172 decisions involving tradeoffs between profits for themselves and pain for either themselves (Figures 1A and 1C) or an anonymous other “receiver” (Figures 1B and 1D) [12]. We separately manipulated whether participants *increased* pain via a motor action (Figures 1A and 1B) or *decreased* pain via a motor action (Figures 1C and 1D). To avoid habituation and sensitization and preserve choice independence, we delivered no money or shocks during the task. Instead, one trial was selected by the computer and implemented at the end of the experiment. Decisions were completely anonymous and confidential with respect to both the receiver and the experimenters. Subjects were made aware of these details.

Thus, our experimental design allowed us to investigate the drugs’ effects on motor impulsivity—i.e., a propensity to respond prematurely before considering the consequences of action [32]—independently from their effects on harm aversion per se. This is important because previous work cannot rule out the possibility that monoamines influence aggression via their evident effects on motor impulsivity [13, 33, 34]. If the link between monoaminergic function and antisocial behavior is mediated by monoaminergic influences on motor impulsivity, then we would expect to see drug effects only in trials where subjects increased harm via action. By contrast, if monoamines influence antisocial behavior through effects on valuation of harmful outcomes, then we would expect to see drug effects in all trials, regardless of action requirements.

### Computational Model of Moral Decision Making

We fit a computational model to subjects’ choice data and examined the effects of citalopram and levodopa on the model parameters. This approach tests the ability of a hypothesized set of cognitive processes to account for all choices rather than only hand-selected aspects of the data [35]. We used a model independently validated in two previous behavioral studies using the same decision task [12]. The model explained the data well, correctly predicting 84% of deciders’ choices in study 1 (95% confidence interval [CI] [83–86]) and 85% of deciders’ choices in study 2 (95% CI [84–86]). The model allows for distinct valuation of harms to self and other and incorporates a factor that accounts for loss aversion for both shocks and money:

$$\Delta V=\left(1-\kappa \right){ℒ}_{m}\Delta m-\kappa {ℒ}_{s}\Delta s$$

$$\kappa =\{\begin{array}{c} \kappa_{self}\;\text{if}\;\text{self}\;\text{trial}\\ \kappa_{other}\;\text{if}\;\text{other}\;\text{trial} \end{array}$$

$${ℒ}_{m}=\{\begin{array}{c} 1\;\text{if}\;\Delta m>0\\ \lambda \;\text{if}\;\Delta m<0 \end{array}$$

$${ℒ}_{s}=\{\begin{array}{c} 1\;\text{if}\;\Delta s<0\\ \lambda \;\text{if}\;\Delta s>0 \end{array},$$

where Δ*V* is the subjective value of switching from the default to the alternative option, and Δ*m* and Δ*s* are the objective differences in money and shocks between the default and alternative options, respectively. Δ*V* is based on a weighted average of these two quantities, where the relative weighting given to Δ*s* is determined by a harm aversion parameter κ. When κ = 0, deciders will accept any number of shocks to increase profits. As κ approaches 1, deciders become maximally harm averse and will pay increasing amounts to avoid a single shock. The setting of κ depends on who is receiving the shocks, where κ_self_ and κ_other_ capture the subjective cost of pain for self and others, respectively. Finally, the objective differences, Δ*m* and Δ*s*, are modulated by a loss aversion parameter λ that captures the difference in the sensitivity of subjective value to gains (increases in money or decreases in shocks) versus losses (decreases in money or increases in shocks) [14]. Trial-by-trial value differences were transformed into choice probabilities using a softmax function [36].

### Drug Effects on Moral Decisions

We previously observed in this exact setting that subjects were hyperaltruistic, in that harm aversion was greater for others than for self [12]. We replicate this effect here in the placebo groups of both studies (study 1: t_(45)_ = −2.08, p = 0.043; study 2: t_(42)_ = −2.37, p = 0.023). Hyperaltruism (computed as the difference in harm aversion for self and others, i.e., κ_other_ − κ_self_) resulted in subjects being willing to pay, on average, an extra 10p per shock to prevent shocks to others, relative to themselves.

Our primary aim was to examine the effects of citalopram and levodopa on harm aversion for self and others (captured by our model’s harm aversion parameters κ_self_ and κ_other_). We formally tested this in an omnibus mixed-effects ANOVA on the harm aversion parameter estimates with shock recipient (self, other) as a within-subjects factor and study and drug as between-subjects factors. We found a significant dissociation in the effects of citalopram and levodopa on harm aversion for self and others (study × drug interaction, F_(1,171)_ = 5.268, p = 0.023).

**Figure 2:**
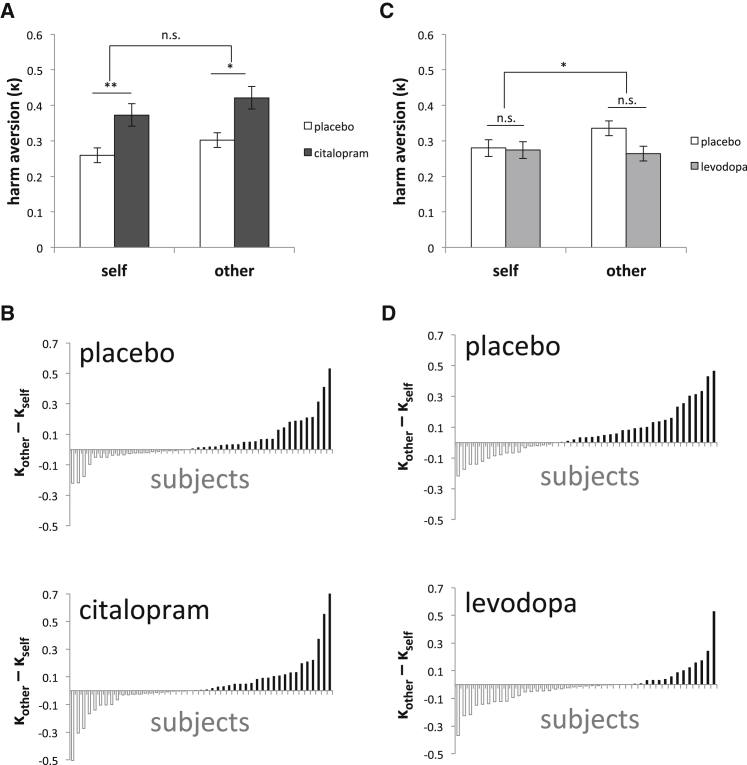
Effects of Citalopram and Levodopa on Harm Aversion and Hyperaltruism in Study 1 and Study 2 (A) In study 1, Citalopram significantly increased harm aversion for self (κ_self_) and others (κ_other_) but did not affect the difference in harm aversion between self and others (i.e., hyperaltruism). (B) Sorted effect sizes of hyperaltruism (defined by taking the difference between κ_other_ and κ_self_) across participants for the placebo and citalopram groups in study 1. Black bars indicate hyperaltruistic subjects, while white bars indicate selfish subjects. (C) In study 2, Levodopa did not affect harm aversion for self or others, but significantly decreased the difference in harm aversion between self and others. (D) Sorted effect sizes of hyperaltruism (κ_other_ − κ_self_) across participants for the placebo and levodopa groups in study 2. ^∗^p < 0.05; ^∗∗^p < 0.01; n.s., not significant. Error bars represent SEM difference between κ_self_ and κ_other_.

Next, we examined separately the drugs’ effects on the harm aversion parameters. Citalopram increased harm aversion both for self and others (main effect of drug, F_(1,87)_ = 7.114, p = 0.009; κ_self_, t_(87)_ = −2.761, p = 0.007; κ_other_, t_(87)_ = −2.240, p = 0.028; Figure 2A). There was no interaction between shock recipient and drug (F_(1,87)_ = 0.016, p = 0.90) indicating that citalopram influenced harm aversion for self and others to the same degree. Correspondingly, subjects on citalopram delivered fewer shocks to themselves and others than those on placebo (main effect of drug, F_(1,87)_ = 6.220, p = 0.015; shocks to self: t_(87) =_ −2.673, p = 0.009; shocks to others: t_(87)_ = −2.353, p = 0.021). The effects of citalopram on harm aversion could not be explained by a reduction in motor impulsivity (Supplemental Information). Strikingly, citalopram nearly doubled the subjective cost per shock, both for self (from 35p to 60p per shock) and others (from 44p to 73p per shock). This resulted in subjects on citalopram choosing to deliver, on average, 30 fewer shocks to themselves and 35 fewer shocks to others over the course of the experiment, relative to subjects on placebo. Levodopa, by contrast, did not significantly affect harm aversion for self or others (κ_self_, t_(42)_ = −0.318, p = 0.75; κ_other_, t_(42)_ = 1.099, p = 0.28).

We then examined the drugs’ effects on hyperaltruism. Planned comparisons indicated that citalopram did not affect hyperaltruism (drug × recipient interaction, F_(1,87)_ = 0.016, p = 0.90; Figure 2B). By contrast, levodopa reduced hyperaltruism (drug × recipient interaction, F_(1,84)_ = 4.358, p = 0.040; Figures 2C and 2D), to the extent that subjects in the levodopa group did not show significantly greater harm aversion for others than for self (t_(42)_ = 0.497, p = 0.622). This resulted in subjects on levodopa choosing to deliver, on average, ten more shocks to the receiver over the course of the experiment, relative to subjects on placebo. Accordingly, subjects tended to deliver fewer shocks to others than themselves on placebo, but not levodopa (drug × recipient interaction, F_(1,84)_ = 3.048, p = 0.084). The omnibus three-way interaction between study, drug, and shock recipient did not reach significance (F_(1,171)_ = 2.066, p = 0.152), leaving open the possibility that citalopram may affect hyperaltruism, albeit to a lesser degree than levodopa. The reduction in hyperaltruism observed following levodopa could not be explained by increased motor impulsivity (Supplemental Information).

### Selectivity of Drug Effects

Importantly, the effects of the drugs on harm aversion could not be attributed to changes in subjective experience of pain, as neither drug affected subjects’ pain thresholds (citalopram: t_(85)_ = −0.4102, p = 0.6827; levodopa: t_(84)_ = −0.0166, p = 0.9868; Figure S1). Moreover, neither drug affected estimates of the loss aversion parameter λ (citalopram: *z =* 1.05, p = 0.295; levodopa: *z =* 1.42, p = 0.157).

We performed additional analyses to confirm the selectivity of our effects. Subjective feeling reports on 16 dimensions, measured pre-task and post-task, did not differ significantly for levodopa versus placebo (Table S1). For citalopram versus placebo, we observed increases in the states “feeble,” “troubled,” and “incompetent,” although none survived multiple comparison correction (Table S1). Nevertheless, the effects of citalopram on harm aversion for self and others remained significant when controlling for these state changes (κ_self_: β = 0.08 ± 0.04, p = 0.047; κ_other_: β = 0.11 ± 0.06, p = 0.049), and none of the mood variables significantly affected harm aversion or interacted with the drug effects (all p > 0.14).

### Evidence for Dose Dependency of Drug Effects

**Figure 3:**
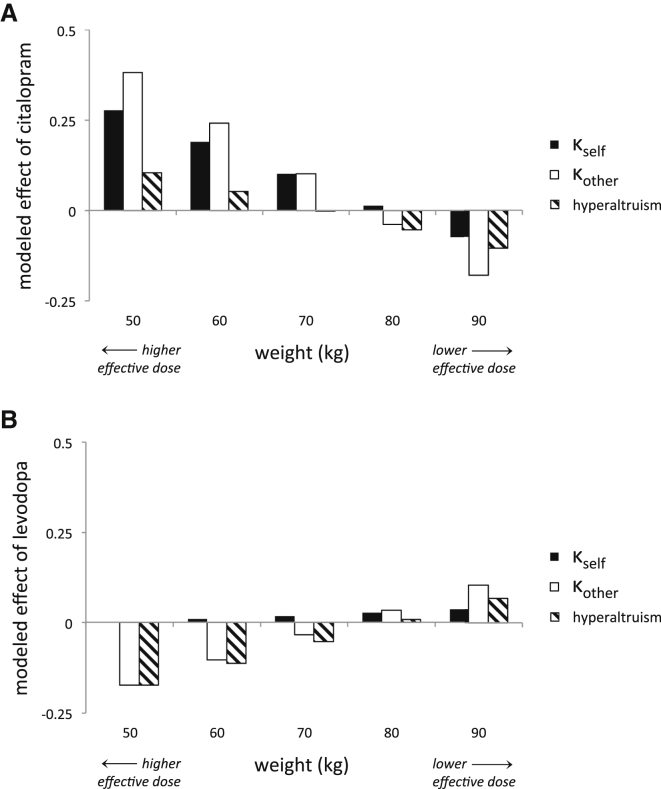
Predictions of Fitted Regression Models of the Interaction of Drug and Effective Dosage on Harm Aversion for Self and Others and Hyperaltruism (A) Citalopram increased harm aversion for self and others relative to placebo, more strongly for subjects with lower body weight (who thus received a higher effective dose). (B) Levodopa reduced hyperaltruism relative to placebo, more strongly for subjects with lower body weight (who thus received a higher effective dose).

We also tested for causality in the drugs’ effects by examining the influence of effective drug dosage (which varied according to subjects’ body weight: 0.31–0.62 mg/kg for citalopram, 1.43–3.35 mg/kg for levodopa). For each drug, we performed a linear regression testing jointly for the effects of drug and the interaction of drug and effective dose, controlling for sex and body mass index, as these factors may themselves be associated with baseline monoaminergic function [37, 38]. For citalopram, this analysis revealed significant effects of drug (κ_self_, t_(81)_ = 2.50, p = 0.014; κ_other_, t_(81)_ = 3.07, p = 0.003) and drug × effective dose (κ_self_, t_(81)_ = −2.03, p = 0.046; κ_other_, t_(81)_ = −2.61, p = 0.011), indicating that subjects receiving a larger effective dose showed a greater effect of citalopram on harm aversion for self and others (Table S2; the model’s account of this is shown in Figure 3A). There was no effect of sex or sex × drug on harm aversion for self or others (all p > 0.39). In a parallel analysis of raw choice data, citalopram reduced the number of shocks delivered to self (t_(82)_ = 2.20, p = 0.031) and others (t_(82)_ = 2.65, p = 0.01) and did so as a function of effective dose (self: t_(82)_ = −1.88, p = 0.064; other: t_(82)_ = −2.28, p = 0.025).

A similar analysis for levodopa revealed significant effects of drug (t_(78)_ = −2.22, p = 0.030) and a trend level interaction between drug and effective dose (t_(78)_ = 1.90, p = 0.060) on hyperaltruism (Table S2; the model’s account of this is shown in Figure 3B), suggesting that subjects receiving a larger effective dose showed a greater effect of levodopa on hyperaltruism. There was no effect of sex or sex × drug on hyperaltruism (all p > 0.45). A corresponding analysis of raw choice data showed that levodopa significantly reduced the difference in shocks delivered to self versus others (t_(79)_ = −2.12, p = 0.038) and tended to do so as a function of effective dose (t_(79)_ = 1.92, p = 0.059).

### Drug Effects on Response Times

**Figure 4:**
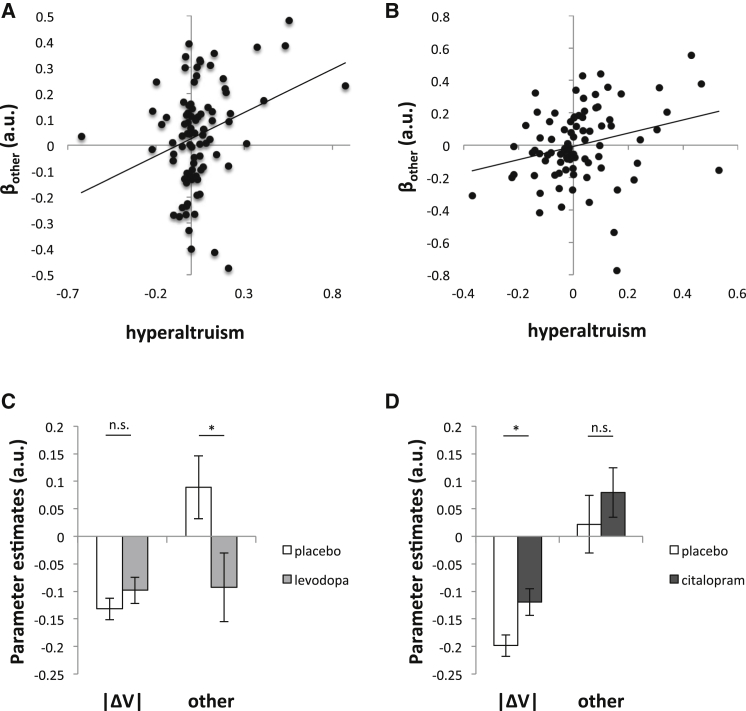
Effects of Hyperaltruism and Drugs on Response Times (A and B) Hyperaltruism (κ_other_ − κ_self_) was positively correlated with slower decisions for others relative to self in study 1 (A) and study 2 (B), as measured by the weight of a general linear model regressor indicating whether the outcome was for other (β_other_). (C) Levodopa reduced slowing associated with deciding for others, relative to self, but did not affect speeding related to value differences (|Δ*V|*). (D) Citalopram reduced speeding related to value differences but did not affect slowing associated with deciding for others, relative to self. ^∗^p < 0.05. Error bars represent SEM. a.u., arbitrary units. See also Table S3.

Neither drug affected overall response times (citalopram: t_(87) =_ 1.32, p = 0.19; levodopa: t_(84)_ = −0.254, p = 0.80). Previously we found that hyperaltruism was related to slower decisions for others relative to self [12]. We replicated this finding here (study 1: r = 0.29, p = 0.006; Figure 4A; study 2: r = 0.27, p = 0.01; Figure 4B). In light of levodopa’s reduction of hyperaltruism, we investigated whether levodopa also reduced slowing for decisions about others. Because the drugs shifted subjects’ indifference points in terms of the amount of money they were willing to sacrifice to avoid pain, which resulted in them making different choices under drug versus placebo conditions, we restricted our analysis to trials near subjects’ indifference points, examining how the drugs modulated the effects of shock recipient, and differences in subjective value between the choice options, on response times (Table S3). An omnibus ANOVA testing a formal dissociation in the drugs’ effects showed a significant interaction between response time component, study, and drug (F_(1,171)_ = 3.57, p = 0.029), indicating dissociable effects of citalopram and levodopa on response times. Levodopa reduced slowing for others (t_(84)_ = 2.15, p = 0.035) without affecting speeding related to subjective value differences (t_(84)_ = −1.09, p = 0.278; Figure 4C). Meanwhile, citalopram reduced speeding related to subjective value differences (t_(87)_ = −2.23, p = 0.028) without affecting slowing for others (t_(87)_ = −0.698, p = 0.487; Figure 4D). A separate analysis including all trials revealed complementary findings (see Supplemental Information).

## Discussion

We combined pharmacological tools with a computational model of harm aversion to show that serotonin and dopamine manifest dissociable neuromodulatory effects on moral decision making. Inhibition of central serotonin reuptake, which increases synaptic serotonin, strongly and selectively increased harm aversion for both self and others. By contrast, increasing central dopamine levels reduced the extent to which people placed others’ welfare before their own. The drugs also had dissociable effects on response times, and their effects on behavior are not explained by changes in motor impulsivity or subjective mood. The drugs’ effects on model parameters were somewhat stronger than their effects on behaviors in aggregate, highlighting the sensitivity of our model-based approach. Overall, our data provide evidence that serotonin and dopamine modulate moral preferences in distinct ways, with ramifications for understanding prosocial behavior and its disruption in psychiatric disorders.

Our data provide the first direct comparison of serotonergic modulation of harm aversion for self and others. Citalopram increased the subjective cost of harm similarly for self and others, suggesting that serotonin influences social behavior through a general effect on integrating aversive and appetitive values rather than a specific effect on social cognition. This explanation also fits with citalopram’s effects on response times. Citalopram reduced a speeding associated with incentive motivation, suggesting that the drug induced a more cautious response disposition, an effect consistent with previous findings [27, 28]. Citalopram also increased negative affect, consistent with serotonin’s putative role in mood [29], although we did not find evidence that mood mediated the drugs’ effects on harm aversion (Supplemental Experimental Procedures). That citalopram had similar effects on harm aversion for self and others is particularly notable given that self-harm is often comorbid with aggressive, antisocial behavior [39] and that people with psychopathy show deficient responses to punishments to self [1]. A goal for future research is delineating the neural circuitry for computing the relative values of harm to self and others and how this circuitry is modulated by serotonin. Reduced harm aversion may be a risk factor for instrumental and reactive aggression that both feature in psychopathy [40], though serotonin is more strongly implicated in the latter [41].

Levodopa reduced hyperaltruism, albeit to a weaker extent than the effect of citalopram on harm aversion. This finding supports a causal link between phasic dopamine hyperactivity and antisocial behavior in humans [10, 11] and is congruent with past work showing levodopa increases selfishness for monetary reward [26]. Our findings are also consistent with a recent report that enhancing tonic dopamine increased inequality aversion [25]. In the current context, increased inequality aversion would reduce prosociality, since hyperaltruism manifests as a preference for inequality in favor of others. We previously suggested that hyperaltruism might be driven by an uncertainty inherent in decisions affecting others [12]. If subjects assume a nonlinear mapping from objective shocks to subjective utility, then uncertainty about the shape of the receiver’s utility function could induce a form of “moral risk aversion” where people err on the side of caution to avoid imposing intolerable costs on others. As uncertainty is associated with slower responding, this explanation gels with observations that hyperaltruism is positively correlated with slower deciding for others relative to oneself [12].

The uncertainty hypothesis suggests a mechanism through which levodopa reduces hyperaltruism and slowing when deciding for others. Dopamine may reduce uncertainty about others’ utilities by reducing the variability of neural representations of others [42]. Such a mechanism could be implemented by dopaminergic modulation of the medial prefrontal cortex (mPFC), a region that encodes uncertainty about others’ intentions [43] and receives dopamine projections [44].

An alternative explanation relates to dopamine’s putative role in safety signaling and active avoidance [20]. Trials where the shocks are assigned to the other rather than oneself could be treated as safety signals, evoking a dopaminergic prediction error that is enhanced by levodopa. Increased dopaminergic tone could then stimulate reward-seeking behavior and increase response vigor [45]. This would result in reduced harm aversion and faster responding when deciding for others relative to oneself. While the uncertainty hypothesis predicts that a prefrontal mechanism would mediate dopamine’s effects on hyperaltruism, the safety signaling mechanism would likely be mediated through the striatum. Neuroimaging studies could help resolve these competing explanations.

We capitalized on variation in subjects’ body weight to examine the potential effects of effective dosage. This analysis suggested that subjects with lower body weight, who thus received a higher effective dose, showed stronger drug effects on moral decision making. An important caveat is that weight may influence baseline monoaminergic function [37, 38]. We attempted to mitigate potential baseline differences by controlling for sex and BMI in our analyses. The observed interactions between drug and body weight could also result from a biphasic dose-response curve that has been observed previously for citalopram and levodopa [46, 47]. Potential dose dependency of our effects could be more optimally addressed in future studies using a within-subjects design with multiple drug doses.

We have shown that some of the most commonly prescribed psychiatric drugs influence moral decisions, raising important questions about the ethics of pharmacological interventions. A single dose of citalopram nearly doubled the amount of money people were willing to pay to avoid harming others, while a single dose of levodopa eliminated a hyperaltruistic tendency to prefer harming oneself over others. However, it is important to stress that these drugs probably have different effects in healthy volunteers compared to patients, and future work could usefully investigate how serotonin and dopamine influence harm aversion in psychiatric disorders with monoaminergic abnormalities. The model-based approach we employ here is a first step in this direction. These methods enabled us to probe the mechanisms driving neuromodulatory effects on choice and also provide an obvious pathway to future work investigating the neural computations supporting prosocial behavior and its impairment in psychiatric disorders.

## Experimental Procedures

### Participants

We recruited healthy participants aged 18–35 years, excluding individuals with a history of psychiatric disorders, cardiac or endocrine disorder, medication or drug use (other than contraceptive pills), or previous allergic reactions to medications, individuals who may be pregnant or are breast feeding, or individuals with >1 year studying psychology. Participants were instructed to avoid taking caffeine on the day of the study, alcohol and pain medication 24 hr before the study, and recreational drugs 7 days prior to participation.

95 participants (47 male, 48 female; mean age = 22.3 ± 3.85) took part in study 1 (citalopram). Two participants did not complete the study due to side effects, and four participants were excluded for not believing there was a real receiver or for not finding the shocks aversive. The final analysis included 89 participants (drug n = 43, placebo n = 46). 92 participants (46 male, 46 female; mean age = 22.3 ± 3.53) took part in study 2 (levodopa). Six participants were excluded for not believing there was a real receiver or for not finding the shocks aversive. The final analysis included 86 participants (drug n = 43, placebo n = 43). In both studies, the drug and placebo groups did not differ significantly in terms of sex, age, education, or social and emotional traits (Table S4).

### Procedure

The two studies were run in parallel at the Wellcome Trust Centre for Neuroimaging in London and were approved by the University College London Research Ethics Committee (4418/003). Participants completed online questionnaires 1 week before the testing session. Two individuals participated in each session. They arrived at staggered times and were led to separate testing rooms without seeing one another.

Upon arrival, participants gave their written informed consent and underwent a medical exam. Participants were randomly assigned to receive either drug or placebo under double-blind conditions. Subjects were unable to distinguish whether they received drug or placebo (citalopram: χ^2^ = 0.36, p = 0.55; levodopa: χ^2 =^ 0, p = 1.0). Subjective state questionnaires were collected at baseline and at three other times during the study. In study 1, participants received either citalopram (30 mg drops, dissolved in orange juice) or placebo (orange juice). In study 2, participants received either levodopa (187.7 mg “Madopar,” comprised of 150 mg levodopa and 37.5 mg benserazide, dissolved in orange juice) or a placebo (vitamin C tablet containing ascorbic acid, lactose, and sucrose, dissolved in orange juice).

The start of the harm aversion task was delayed to coincide with peak drug absorption (3 hr or 60 min after drug administration for citalopram and levodopa, respectively). During the waiting period, participants completed additional questionnaires. Before the task, participants completed a role randomization procedure (described in detail elsewhere [12]) that ostensibly assigned them and the other participant to different roles. Next, they completed a pain thresholding procedure [31] followed by the harm aversion task [12] (Supplemental Experimental Procedures). Next, participants completed a learning task (data not shown). After this, the outcome of one trial was delivered. Before departing, participants completed a debriefing questionnaire.

### Data Analysis

We used a model derived from previous studies using the harm aversion task [12] that explained choices in terms of the value difference (Δ*V*) between the default and alternative options. Trial-by-trial value differences were transformed into choice probabilities using a softmax function [36]:

$$P\;\left(choose\;alternative\right)=\left(\frac{1}{1+e^{-\gamma \Delta V}}\right)\left(1-2\varepsilon \right)+\varepsilon ,$$

where γ is a subject-specific inverse temperature parameter that characterizes the sensitivity of choices to *ΔV*, and ε is an irreducible noise parameter that captures choice noisiness resulting from factors independent of *ΔV* (such as inattention). We optimized subject-specific parameters across trials using nonlinear optimization implemented in MATLAB (MathWorks) for maximum likelihood estimation. Parameters were estimated individually for each subject, and summary statistics were calculated from these parameter estimates at the group level, treating each parameter estimate as a random effect [48]. We tested the effects of the drugs on model parameters using t tests (for normally distributed parameters) and nonparametric Wilcoxon rank-sum tests (for non-normally distributed parameters).

Response times were log transformed and *Z* scored prior to analysis. We modeled response times using a general linear model validated in previous studies using the harm aversion task [12]. We analyzed the first 88 trials in the task, as these trials were identical for all participants. The model contained regressors indicating whether the outcome was for self or other (*other*); the difference in shocks between the default and alternative options (*Δs*); the difference in money between the default and alternative options (*Δm*); the maximum number of shocks that could be delivered (*s*_*max*_); the interaction between the difference in money and difference in shocks between the default and alternative options (*Δs* × *Δm*); the interaction between the difference in shocks between the default and alternative options and whether the outcome was for self or other (*Δs* × *other*); and a constant. Summary statistics were calculated from regressor beta weights at the group level, treating each beta weight as a random effect [48]. We tested the effects of the drugs on beta weights using t tests.

We performed a separate analysis restricted to trials near subjects’ indifference points (i.e., for trials whose shock and money amounts created an indifference point that was within ±0.2 units of the subject’s own indifference point). We modeled response times using a general linear model containing regressors indicating whether the outcome was for self or other (*other*); the unsigned difference in subjective value between the two choice options, which was constructed individually for each subject using their model parameters (|*ΔV*|); and a constant term. We tested the effects of the drugs on beta weights using t tests.

## Author Contributions

M.J.C., J.Z.S., Z.K.-N., P.D., and R.J.D. designed the research. J.Z.S., G.S., O.T.O., J.M.G.-R., and C.F. performed the research. M.J.C., J.Z.S., Z.K.-N., J.M.G.-R., and C.F. analyzed the data. All authors contributed to writing the paper.

## Supplemental Information

Document S1. Supplemental Results, Supplemental Experimental Procedures, Figure S1, and Tables S1–S4

Document S2. Article plus Supplemental Information
