# Supplementary material for: Dissociable Effects of Serotonin and Dopamine on the Valuation of Harm in Moral Decision Making
Source: Curr Biol. 2015 Jul 20;25(14):1852–9. doi: 10.1016/j.cub.2015.05.021 (PMC4518463; doi:10.1016/j.cub.2015.05.021)
Supplement: Document S1. Supplemental Results, Supplemental Experimental Procedures, Figure S1, and Tables S1–S4 [file mmc1.pdf]

Current Biology

Supplemental Information

Dissociable Effects of Serotonin and Dopamine on the Valuation of Harm in Moral Decision Making

Molly J. Crockett, Jenifer Z. Siegel, Zeb Kurth-Nelson, Olga T. Ousdal, Giles Story, Carolyn Frieband, Johanna M. Grosse-Rueskamp, Peter Dayan, and Raymond J. Dolan

Supplemental Data

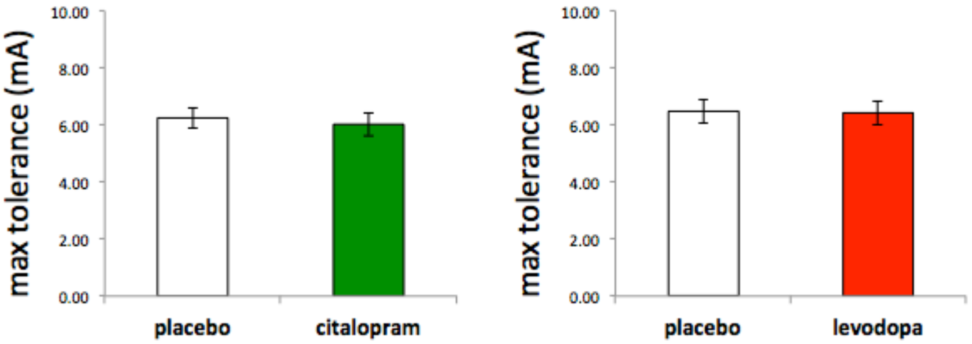

Figure S1, related to Figure 1. Subjects' pain thresholds (the level of stimulation, in mA, corresponding to a 10/10 "intolerable" rating on a subjective pain scale) did not differ between the drug and placebo groups in study 1 (left panel) or study 2 (right panel). Error bars represent SEM. mA, milliamps.

Subjective state questionnaire	Placebo change, study 1	Citalopram change, study 1	P-value, study 1	Placebo change, study 2	Levodopa change, study 2	P-value, study 2
Alert 0 – drowsy 10	-1.51	-0.92	0.23	-0.62	-0.96	0.49
Calm 0 – excited 10	1.15	0.89	0.61	1.44	1.16	0.60
Strong 0 – feeble 10	-0.65	0.40	0.01	0.03	-0.23	0.49
Muzzy 0 – clear headed 10	0.39	0.20	0.70	0.34	-0.33	0.17
Coordinated 0 – clumsy 10	-0.40	-0.42	0.95	-0.15	0.31	0.22
Lethargic 0 – energetic 10	1.15	0.54	0.19	0.96	0.35	0.24
Contented 0 – discontented 10	0.13	0.63	0.20	0.64	0.60	0.88
Troubled 0 – tranquil 10	-0.31	-1.67	0.01	-0.35	-0.69	0.40
Slow 0 – quick witted 10	0.67	0.20	0.21	0.52	0.00	0.28
Tense 0 – relaxed 10	-1.79	-1.58	0.70	-1.35	-1.19	0.73
Attentive 0 – dreamy 10	-1.29	-0.59	0.12	-0.66	-0.24	0.29
Incompetent 0 – proficient 10	0.28	-0.51	0.04	0.36	-0.11	0.29
Happy 0 – sad 10	-0.30	0.32	0.11	0.23	0.36	0.65
Antagonistic 0 – friendly 10	-0.10	-0.75	0.06	-0.39	-0.79	0.41
Interested 0 – bored 10	-0.17	-0.22	0.86	0.28	0.10	0.53
Withdrawn 0 – sociable 10	0.09	0.00	0.79	0.36	0.00	0.46

Table S1, related to Figure 2. Scores represent differences in ratings in a subjective state questionnaire between baseline (before placebo or drug administration) and the start of the task. Questions were answered by marking a point on a line and responses were converted to a 0-10 scale. P-values are not corrected for multiple comparisons. Three variables showed differences (p

< 0.05) between placebo and citalopram (citalopram made subjects feel more feeble, troubled, and incompetent, relative to placebo). None of the observed differences survived correction for multiple comparisons (Bonferroni corrected p = 0.003). None of the variables showed significant differences between placebo and levodopa.

Study 1 (citalopram)						
Regressor	κ_{self}	sh_{self}	κ_{other}	sh_{other}	hyperaltruism	$sh_{self-oth}$
drug	0.70**	374.06**	1.07***	541.30***	-0.37	-167.24
	(0.28)	(170.29)	(0.35)	(204.56)	(0.26)	(137.02)
male	-0.01	-0.36	-0.08	11.86	-0.07	-47.98
	(0.08)	(50.54)	(0.10)	(60.71)	(0.08)	(40.67)
weight	0.002	3.47	0.006	4.58	-0.002	-1.11
	(0.005)	(2.95)	(0.006)	(3.56)	(0.004)	(2.38)
BMI	-0.0003	1.18	-0.007	2.87	-0.01	-1.69
	(0.01)	(8.19)	(0.02)	(9.85)	(0.01)	(6.60)
drug * male	0.04	67.67	0.04	57.76	0.001	9.91
	(0.10)	(59.20)	(0.12)	(71.12)	(0.09)	(47.63)
drug* weight	-0.01**	-4.96*	-0.01**	-7.23**	0.01	2.27
	(0.004)	(2.64)	(0.005)	(3.17)	(0.004)	(2.12)
constant	0.15	93.82	0.11	-71.10	0.34	164.92
	(0.20)	(136.31)	(0.25)	(163.74)	(0.20)	(109.68)
**Study 2 (levodopa)**						
Regressor	κ_{self}	sh_{self}	κ_{other}	sh_{other}	hyperaltruism	$sh_{self-oth}$
drug	-0.02	8.50	-0.46	261.70	-0.45**	-253.19**
	(0.24)	(161.51)	(0.31)	196.05	(0.20)	(120.70)
male	-0.04	7.54	0.001	-10.92	0.04	18.46
	(0.07)	(46.28)	(0.09)	(56.18)	(0.06)	(34.30)
weight	0.001	0.38	-0.004	2.59	-0.005	-2.22
	(0.003)	(1.98)	(0.004)	(2.41)	(0.003)	(1.47)
BMI	-0.0002	0.22	0.0001	0.48	0.001	-0.26
	(0.003)	(1.72)	(0.003)	(2.09)	(0.01)	(1.28)
drug * male	-0.06	43.15	-0.12	87.32	-0.06	-44.16
	(0.09)	(60.54)	(0.12)	(73.49)	(0.08)	(44.87)
drug* weight	0.001	-0.67	0.01	-4.28	0.006*	3.61*
	(0.004)	(2.54)	(0.005)	(3.08)	(0.003)	(1.88)
constant	0.25	372.76	0.58	207.05	0.32	165.71

Table S2, related to Figure 3. Mean parameter estimates for effects of drug, sex, and effective dose (body weight) on harm aversion for self and others, total shocks delivered to self and others,

hyperaltruism, and the difference in total shocks delivered to self vs. others; SEMs in parentheses. ***p < 0.01, **p < 0.05, *p < 0.10

Regressor	Study 1			Study 2		
	**citalopram**	**placebo**	**p-value**	**levodopa**	**placebo**	**p-value**
$\lvert \Delta V \rvert$	-0.12	-0.19	0.03	-0.10	-0.13	0.28
	(0.02)	(0.02)		(0.02)	(0.02)	
other	0.08	0.03	0.49	-0.09	0.09	0.03
	(0.04)	(0.05)		0.06	(0.06)	
constant	-0.34	-0.23	0.18	-0.31	-0.39	0.31
	(0.05)	(0.05)		(0.06)	(0.05)	

Table S3, related to Figure 4. Mean parameter estimates for effects of unsigned value difference ($\lvert \Delta V \rvert$) and shock recipient (*other*) on response times; SEMs in parentheses. P-values indicate the results of unpaired *t*-tests comparing parameter estimates for drug and placebo groups.

Study 1	Placebo (N=46)	Citalopram (N=43)	p-value
**Age (SD)**	22.30 (3.140)	22.47 (4.522)	0.845
**Gender %**			0.924
Male	22 (47.8%)	21 (48.8%)	
Female	24 (52.2%)	22 (51.2%)	
**Education (SD)**	4.28 (1.452)	3.84 (1.511)	0.156
**Behavioural Activation Scale (SD)**	41.59 (4.665)	40.44 (4.339)	0.235
**Behavioural Inhibition Scale (SD)**	20.93 (4.030)	22.21 (2.875)	0.091
**Interpersonal Reactivity Index (SD)**	78.61 (10.472)	78.26 (9.878)	0.871
**Altruism Scale (SD)**	60.61 (6.275)	59.95 (5.884)	0.616
**Social Desirability Scale (SD)**	39.59 (7.776)	40.95 (6.222)	0.365
**Psychopathy Scale (SD)**	121.87 (23.210)	114.72 (22.230)	0.142
**Personality Belief Questionnaire (SD)**	143.61 (35.642)	138.95 (29.105)	0.503
**Personality Inventory for DSM-5 (SD)**	49.11 (9.823)	48.35 (8.685)	0.701
**Inventory of Interpersonal Problems (SD)**	69.33 (16.075)	69.09 (14.044)	0.942

Study 2	Placebo (N=43)	Levodopa (N=43)	p-value
**Age (SD)**	22.14 (3.060)	22.56 (3.978)	0.586
**Gender %**			0.388

Male	19 (44.2%)	23 (53.5%)	
Female	24 (55.9%)	20 (46.5%)	
**Education (SD)**	4.40 (1.330)	4.21 (1.283)	0.511
**Behavioural Activation Scale (SD)**	40.86 (4.533)	39.37 (4.986)	0.151
**Behavioural Inhibition Scale (SD)**	21.19 (2.780)	20.21 (3.967)	0.190
**Interpersonal Reactivity Index (SD)**	77.16 (9.978)	74.79 (12.881)	0.343
**Altruism Scale (SD)**	61.28 (4.763)	60.81 (11.289)	0.804
**Social Desirability Scale (SD)**	40.91 (6.328)	40.70 (7.163)	0.886
**Psychopathy Scale (SD)**	126.56 (22.255)	124.19 (29.436)	0.674
**Personality Belief Questionnaire (SD)**	146.05 (34.239)	148.16 (34.259)	0.775
**Personality Inventory for DSM-5 (SD)**	48.95 (10.415)	47.14 (8.983)	0.390
**Inventory of Interpersonal Problems (SD)**	71.49 (17.644)	64.35 (19.303)	0.077

Table S4. Demographic characteristics of drug and placebo groups in studies 1 and 2

Supplemental results: motor impulsivity, related to Figure 2

To examine whether the observed serotonergic modulation of harm aversion could be explained by reduced motor impulsivity, we fit separate harm aversion parameters for self and others on trials where action increased harm and trials where inaction increased harm, and tested the effects of citalopram on harm aversion in a mixed ANOVA with shock recipient (self, other) and action (increase, decrease) as within-subjects factors, and drug (citalopram, placebo) as a between-subjects factor. This analysis ruled out the possibility that citalopram increased harm aversion by reducing impulsivity, as we observed a main effect of citalopram on harm aversion ($F_{(1,87)} = 7.910$, $p = 0.006$) but no interaction between drug and action ($F_{(1,87)} = 0.042$, $p = 0.838$).

The reduction in hyperaltruism observed following levodopa could not be explained by increased motor impulsivity. We fit separate harm aversion parameters for self and others on trials where actions increased and decreased harm and computed hyperaltruism separately for these increasing and decreasing trials, as in the above analysis with citalopram. We observed a main effect of levodopa on hyperaltruism ($F_{(1,84)} = 4.104$, $p = 0.046$) but no interaction between drug and action ($F_{(1,84)} = 1.126$, $p = 0.292$).

Supplemental results: response times, related to Figure 4

We were able to examine whether the drugs moderated the influences of harm, profits, and shock recipient on response times because these factors were varied independently across trials. We describe here the results of an analysis that includes all trials, i.e., not just those around subjects' indifference points. In a general linear model testing the effects of levodopa on changes in response times related to harm, profits, shock recipient, and interactions between these factors, the effect of levodopa on slowing for others was not significant ($t_{(84)}$ = -1.19, p = 0.236). However, we observed an effect of levodopa on the speed of decisions that specifically involved increasing harm to others. On those trials, levodopa reduced slowing for others relative to self as a function of increasing harm magnitude ($t_{(81)}$ = -2.09, p = 0.042, corrected for multiple comparisons). Citalopram did not affect the speed of decisions involving increasing harm to others ($t_{(75)}$ = 0.27, p = 0.786).

The effect of citalopram on response times is consistent with a role for serotonin in aversive processing. Subjects were generally faster when responding resulted in greater profit ($\beta_{\Delta m}$ = -0.04 $\pm$ 0.002, $t_{(88)}$ = -18.12, p = $2e^{-31}$). This incentive-induced speeding was reduced when responding also resulted in relatively greater harm (i.e., we observed an interaction between profit and harm on response times; $\beta_{\Delta s*\Delta m}$ = 0.002 $\pm$ $4e^{-4}$, $t_{(88)}$ = 3.97, p = 0.0001). This latter effect, whereby the presence of harm reduced incentive motivation, was enhanced by citalopram ($t_{(42)}$ = -2.70, p = 0.008), in line with previous findings implicating serotonin in behavioral inhibition in the face of aversive expectations, i.e., aversive Pavlovian-to-instrumental transfer [S1, S2].

It is worth noting that the effects of levodopa and citalopram on response times when considering all trials together differed slightly from the drugs' effects on response times when considering only trials around subjects' indifference points. However, these different analytical approaches, which are influenced by ceiling and floor effects in different ways, revealed effects that point in the same direction. In both cases, levodopa reduced components of slowing when deciding for others relative to oneself, whilst citalopram enhanced aspects of behavioral inhibition. Further research is needed to tease apart the finer aspects of how levodopa and citalopram influence the speed of decisions involving harm to self or others.

Supplemental Experimental Procedures

Pain thresholding procedure

Participants underwent individual pain titration procedures with a Digitimer DS5 electric stimulator. Following a brief overview of the equipment and titration process, two electrodes

were placed on the back of the participant's left wrist. Titration began with a low-intensity electric shock (0.1 mA) and subjects were asked to rate their experience of pain on an 11-point scale (ranging from 0 = no pain to 10 = intolerable). The initial rating was followed by a series of shocks, either increasing or decreasing in small milliamp increments with a 3:1 ratio. Subjective ratings of pain were collected after each shock until a rating of 10 was reached, which was recorded as the maximum threshold. Titration was repeated three times for every participant.

Next, we fit a sigmoid function to a series of shocks, allowing us to estimate the current-to-rating response curve. To do this, we generated a series of seven shocks that ranged from 40 to 100 percent of the subject's maximum threshold in 10% increments. Each of the seven shock intensities was delivered three times in random order. From the derived function we estimated the current intensity that corresponded to each participant's level 8 pain experience. The stimulation level corresponding to a subjective level 8 was used to deliver shocks based on the outcomes in the decision-making task. The titration procedure also served to provide an explicit experience of the aversive stimulus, thus allowing subjects to make meaningful judgments and decisions throughout the task.

We note that phasic pain stimulation is widely used in research settings [S3, S4]. We used electric stimulation because it is consistently judged as aversive across participants and remains so throughout the course of an experiment. Furthermore, electric shocks are not common stimuli in daily life; thus, no existing monetary value could be associated with them.

Moral decision task

All participants were instructed that they were assigned to the role of decider and that the other participant in the session was assigned to the role of receiver. In fact all participants played the role of decider. Behavior in the placebo conditions of the current study was no different from that in previous studies using this paradigm that did not use deception [S5]. We excluded participants who did not believe there was another participant present in the lab; the proportion of participants excluded for this reason was no different from that in previous studies that did not use deception [S5].

The task contained a total of 172 trials. Each trial consisted of a choice between a default amount of shocks and money and an alternative amount of shocks and money. The first 88 trials were a fixed set of choices that were presented to all subjects. To create these we first created a set of 22 trials, each containing a pair of choices that matched the indifference point of a specific κ value (where κ is a harm aversion parameter in our computational model of decision-making describing the exchange rate between money and pain). Across a set of κ values evenly

distributed across the range of κ values observed in previous studies [S5] (from 0 to 1), for each κ value we generated 10,000 random pairs of positive shock movements Δs and positive money movements Δm and selected the pair [Δs, Δm] closest to the indifference point of that κ value.

Next, these optimized pairs [Δs, Δm] were transformed into choices containing default amounts of shocks and money (s_d and m_d) and alternative amounts of shocks and money (s_a and m_a) as follows: s_d was a positive integer between 0 and 20, randomly drawn from a uniform discrete distribution with the constraint that $0 < s_d + \Delta s < 20$. Similarly, m_d was a positive number between 0 and 20, randomly drawn from a uniform discrete distribution, rounded to the nearest 10th and constrained such that $0 < m_d + \Delta m < 20$. s_a and m_a were then set by adding Δs and Δm to s_d and m_d, respectively. Following this process we had a set of 22 "increase" trials where $s_a > s_d$ and $m_a > m_d$.

We next created a set of 22 mirror-image "decrease" trials by swapping the values of s_a and s_d, and likewise m_a and m_d. Each trial was then presented twice, once in the "self" condition and once in the "other" condition, for a total of 88 trials.

The second 84 trials contained 44 trials generated in a manner similar to the first 88 trials, interspersed with an additional 40 trials that were individually tailored to each subject. These latter trials were included to provide more precise estimates of subjects' harm aversion parameters (κ_i) across the four experimental conditions (self-increase, self-decrease, other-increase, other-decrease). To create these trials, we fit a computational model (described below) to the first 88 trials. We then used the harm aversion parameter estimates derived from the model to create a set of 10 trials within +/- 0.1 units of subjects' κ_i estimate for each experimental condition. These were created by selecting a κ value within +/- 0.1 units of κi, generating 10,000 random pairs of positive shock movements Δs and positive money movements Δm, and then selecting the pair [Δs, Δm] closest to the indifference point of that κ value. We note that previous studies using variants of this paradigm have shown that decision-making in this task is unaffected by whether subjects receive a fixed set of trials or an individually tailored set of trials [S5]. Furthermore, subjects' parameter estimates in the placebo condition of the current study were similar to those observed in previous studies using variants of this paradigm.

Experimental design

We employed a between-subjects design, whereby each subject took part in a single testing session where they were randomly assigned to receive either drug or placebo before completing the harm aversion task. We note that a more powerful approach to investigate drug effects on behavior is to use a within-subjects design, whereby each subject receives both drug and placebo

across two testing sessions. Within-subjects designs allow for a more straightforward interpretation of how effective dosage and subjective state moderate the drug effects, as each subject's change in parameter estimates between the drug and placebo sessions can be plotted against each subject's body weight and subjective state. Unfortunately, a within-subjects design was not possible for the present study due to features of our task design, which ensures that choices are made within an incentive-compatible framework. This is because if subjects learn in session 1 that they will be trading money for pain, they would have an incentive in the session 2 to report a lower pain threshold than their true threshold, in order to maximize their profits. Because it was essential for our study that choices were incentive-compatible, we were limited to a between-subjects design. As such, we had to resort to modelling the effects of effective dosage and subjective state using linear regressions as reported here.

Subjective state analysis

We collected subjective feeling reports on 16 dimensions before and after the task to measure potential drug effects on subjective mood. We did not have any a priori hypotheses about whether citalopram or levodopa would affect subjective mood, as previous studies using identical doses of these drugs in a similar study population did not find drug effects on subjective mood states [S6–S9]. However, we found that citalopram increased subjective feelings of being troubled, feeble and incompetent (though these effects did not survive correction for multiple comparisons). To investigate the possibility that mood changes induced by citalopram mediated the effects of the drug on harm aversion, we tested whether the drug effects on harm aversion remained significant when controlling for mood changes, and whether mood changes interacted with the effect of drug on harm aversion. Our results do not suggest that mood changes mediated the effects of citalopram on harm aversion, as the effect of citalopram on harm aversion remained significant after controlling for changes in mood, and there were no significant interactions between drug and mood changes.

If anything, the mood changes induced by citalopram could have masked the drug's effect on harm aversion, particularly for others. Many studies have shown that prosocial behavior is related to positive mood [S10–S12], and prosocial behavior is negatively associated with feelings of incompetence and self-efficacy [S13, S14]. Thus, previous work predicts that feelings of being troubled, feeble and incompetent should reduce subjects' willingness to sacrifice money to reduce the pain of others, whereas citalopram increased this behavior. Although an investigation of how subjective feeling states influence harm aversion for self and others was beyond the scope of the current study, this remains an important topic for future research.

Supplemental References

S1. Crockett, M. J., Clark, L., Apergis-Schoute, A. M., Morein-Zamir, S., and Robbins, T. W. (2012). Serotonin modulates the effects of Pavlovian aversive predictions on response vigor. Neuropsychopharmacology *37*, 2244–2252.

S2. Geurts, D. E. M., Huys, Q. J. M., Ouden, H. E. M. den, and Cools, R. (2013). Serotonin and Aversive Pavlovian Control of Instrumental Behavior in Humans. J. Neurosci. *33*, 18932–18939.

S3. Vlaev, I., Seymour, B., Dolan, R. J., and Chater, N. (2009). The price of pain and the value of suffering. Psychol. Sci. *20*, 309–317.

S4. Story, G. W., Vlaev, I., Seymour, B., Winston, J. S., Darzi, A., and Dolan, R. J. (2013). Dread and the Disvalue of Future Pain. PLoS Comput Biol *9*, e1003335.

S5. Crockett, M. J., Kurth-Nelson, Z., Siegel, J. Z., Dayan, P., and Dolan, R. J. (2014). Harm to others outweighs harm to self in moral decision making. Proc. Natl. Acad. Sci. *111*, 17320–17325.

S6. Crockett, M. J., Clark, L., Hauser, M. D., and Robbins, T. W. (2010). From the Cover: Serotonin selectively influences moral judgment and behavior through effects on harm aversion. Proc. Natl. Acad. Sci. *107*, 17433–17438.

S7. Guitart-Masip, M., Chowdhury, R., Sharot, T., Dayan, P., Duzel, E., and Dolan, R. J. (2012). Action controls dopaminergic enhancement of reward representations. Proc. Natl. Acad. Sci. *109*, 7511–7516.

S8. Chamberlain, S. R., Müller, U., Blackwell, A. D., Clark, L., Robbins, T. W., and Sahakian, B. J. (2006). Neurochemical Modulation of Response Inhibition and Probabilistic Learning in Humans. Science *311*, 861–863.

S9. Sharot, T., Guitart-Masip, M., Korn, C. W., Chowdhury, R., and Dolan, R. J. (2012). How Dopamine Enhances an Optimism Bias in Humans. Curr. Biol. *22*, 1477–1481.

S10. Carlson, M., Charlin, V., and Miller, N. (1988). Positive mood and helping behavior: A test of six hypotheses. J. Pers. Soc. Psychol. *55*, 211–229.

S11. Rand, D. G., Kraft-Todd, G., and Gruber, J. (2015). The Collective Benefits of Feeling Good and Letting Go: Positive Emotion and (dis)Inhibition Interact to Predict Cooperative Behavior. PLoS ONE *10*, e0117426.

S12. Isen, A. M., and Levin, P. F. (1972). Effect of feeling good on helping: Cookies and kindness. J. Pers. Soc. Psychol. *21*, 384–388.

S13. Caprara, G. V., Alessandri, G., and Eisenberg, N. (2012). Prosociality: The contribution of traits, values, and self-efficacy beliefs. J. Pers. Soc. Psychol. *102*, 1289–1303.

S14. Caprara, G. V., Alessandri, G., Di Giunta, L., Panerai, L., and Eisenberg, N. (2010). The contribution of agreeableness and self-efficacy beliefs to prosociality. Eur. J. Personal. *24*, 36–55.
