# Supplementary material for: Dissociable Effects of Serotonin and Dopamine on the Valuation of Harm in Moral Decision Making
Source: Curr Biol. 2015 Jul 20;25(14):1852–9. doi: 10.1016/j.cub.2015.05.021 (PMC4518463; doi:10.1016/j.cub.2015.05.021)
Supplement: Document S2. Article plus Supplemental Information [file mmc2.pdf]

Report

Current Biology

Dissociable Effects of Serotonin and Dopamine on the Valuation of Harm in Moral Decision Making

Highlights

- Serotonin and dopamine had distinct effects on decisions to harm self versus others
- Computational models revealed a hyperaltruistic preference to harm self over others
- Pharmacological enhancement of serotonin increased harm aversion for self and others
- Pharmacological enhancement of dopamine reduced hyperaltruism

Authors

Molly J. Crockett, Jenifer Z. Siegel, Zeb Kurth-Nelson, ..., Johanna M. Grosse-Rueskamp, Peter Dayan, Raymond J. Dolan

Correspondence

molly.crockett@psy.ox.ac.uk

In Brief

Crockett et al. pharmacologically manipulated serotonin and dopamine levels in healthy participants to investigate the neuromodulation of decisions to harm self and others. Enhancing serotonin function increased harm aversion for self and others, while enhancing dopamine function reduced a hyperaltruistic preference to harm oneself over others.

Crockett et al., 2015, Current Biology *25*, 1852–1859
July 20, 2015 ©2015 The Authors
http://dx.doi.org/10.1016/j.cub.2015.05.021

Current Biology
Report

Dissociable Effects of Serotonin and Dopamine on the Valuation of Harm in Moral Decision Making

Molly J. Crockett,[1,2,*] Jenifer Z. Siegel,[1] Zeb Kurth-Nelson,[2,3] Olga T. Ousdal,[2,4] Giles Story,[2] Carolyn Frieband,[2] Johanna M. Grosse-Rueskamp,[2] Peter Dayan,[5] and Raymond J. Dolan[2,3]
[1]Department of Experimental Psychology, University of Oxford, 9 South Parks Road, Oxford OX1 3UD, UK
[2]Wellcome Trust Centre for Neuroimaging, University College London, 12 Queen Square, London WC1N 3BG, UK
[3]Max Planck-UCL Centre for Computational Psychiatry and Ageing, University College London, 12 Queen Square, London WC1N 3BG, UK
[4]Department of Radiology, Haukeland University Hospital, Jonas Lies vei 65, 5021 Bergen, Norway
[5]Gatsby Computational Neuroscience Unit, University College London, Alexandra House, 17 Queen Square, London WC1N 3AR, UK
*Correspondence: molly.crockett@psy.ox.ac.uk
http://dx.doi.org/10.1016/j.cub.2015.05.021
This is an open access article under the CC BY-NC-ND license (http://creativecommons.org/licenses/by-nc-nd/4.0/).

SUMMARY

An aversion to harming others is a core component of human morality and is disturbed in antisocial behavior [1–4]. Deficient harm aversion may underlie instrumental and reactive aggression, which both feature in psychopathy [5]. Past work has highlighted monoaminergic influences on aggression [6–11], but a mechanistic account of how monoamines regulate antisocial motives remains elusive. We previously observed that most people show a greater aversion to inflicting pain on others than themselves [12]. Here, we investigated whether this hyperaltruistic disposition is susceptible to monoaminergic control. We observed dissociable effects of the serotonin reuptake inhibitor citalopram and the dopamine precursor levodopa on decisions to inflict pain on oneself and others for financial gain. Computational models of choice behavior showed that citalopram increased harm aversion for both self and others, while levodopa reduced hyperaltruism. The effects of citalopram were stronger than those of levodopa. Crucially, neither drug influenced the physical perception of pain or other components of choice such as motor impulsivity or loss aversion [13, 14], suggesting a direct and specific influence of serotonin and dopamine on the valuation of harm. We also found evidence for dose dependency of these effects. Finally, the drugs had dissociable effects on response times, with citalopram enhancing behavioral inhibition and levodopa reducing slowing related to being responsible for another's fate. These distinct roles of serotonin and dopamine in modulating moral behavior have implications for potential treatments of social dysfunction that is a common feature as well as a risk factor for many psychiatric disorders.

RESULTS

Many aspects of the way the human brain carries out the computations essential for healthy social interactions remain unclear. Overlapping neural representations of the value of one's own and others' outcomes are a central component of empathy [15] and prosocial behavior [16], suggesting that attention be paid to neural systems involved in valuation. Central among these are the neuromodulators serotonin and dopamine. Indeed, many psychiatric disorders associated with monoaminergic abnormalities feature social dysfunction [1, 11, 17], and interactions between serotonin and dopamine have been implicated in impulsive aggression [18] and psychopathy [19]. However, previous studies have primarily examined how these neuromodulators influence the valuation of outcomes for oneself [20, 21]. How these systems influence the valuation of others' outcomes—particularly harmful ones—is not well understood. A monoaminergic influence on the valuation of harm to others may explain the link between monoamines and aggression that has been observed across species [6–11]. Prior work suggests an influence of serotonin on harm aversion. Deciding whether to harm others engages brain regions densely innervated by serotonin [8, 22, 23], and manipulating serotonin function influences the expression of harm aversion in hypothetical moral judgments [24], though these are not necessarily predictive of real moral decisions [3]. A role for dopamine in harm aversion is less clear. Although biomarkers of hyperactive mesolimbic dopamine function in humans correlate with trait aggression [10] and impulsive-antisocial psychopathic traits [11, 19], direct evidence supporting a causal influence of dopamine on human antisocial behavior is sparse. Previous studies have shown dopaminergic effects on economic decisions [25, 26], but existing economic models are poor predictors of moral decisions concerning harm to others [12].

We recently developed a method for quantifying how people value the pain of others relative to their own pain and observed that most people were "hyperaltruistic," requiring more financial compensation to inflict pain on a stranger than themselves [12]. Here, we investigated how serotonin and dopamine modulate hyperaltruism and the valuation of harm to self and others. In light of past studies suggesting serotonin enhances non-social aversive processing [27–29], we predicted that enhancing serotonin

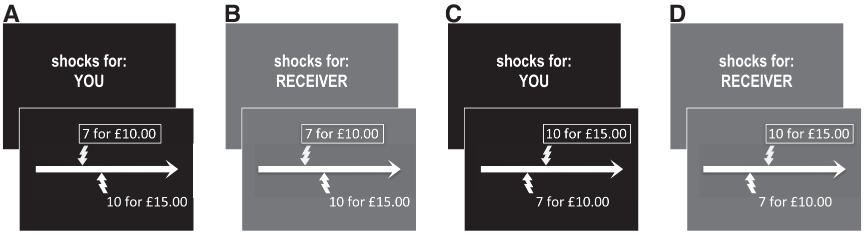

Figure 1. Experimental Design

In each trial, deciders chose between less money and fewer shocks versus more money and more shocks.

(A–D) The money was always for the decider, but in half the trials, the shocks were allocated to the decider (A and C), and, in the other half, the shocks were allocated to the receiver (B and D). In all trials, if the decider failed to press a key within 6 s, the highlighted default (top) option was registered; if the decider pressed the key, the alternative (bottom) option was highlighted and registered instead. In half the trials, the alternative option contained more money and shocks than the default (A and B), so action resulted in greater harm and profit. In the other half, the alternative option contained less money and shocks than the default (C and D), so inaction resulted in greater harm and profit.

function would increase harm aversion for both oneself and others. Meanwhile, given previous work showing positive correlations between high mesolimbic dopaminergic tone and antisocial behavior [10, 11], we predicted that enhancing dopamine function would reduce a hyperaltruistic tendency to prefer harming oneself over harming others.

We tested these hypotheses using double-blind pharmacological manipulations of serotonin and dopamine in subjects performing a moral decision task that allowed us to quantify harm aversion for self and others [12]. In study 1, 89 healthy volunteers received either placebo or 30 mg of the selective serotonin reuptake inhibitor citalopram, which enhances serotonin neurotransmission by blocking its reuptake and prolonging its actions in the synapse [30]. In study 2, 86 healthy volunteers received either placebo or 150 mg of the dopamine precursor levodopa, which elevates central dopamine levels [30]. The decision task was timed to coincide with peak drug absorption.

We first used a standard procedure to determine each subject's pain threshold for an electrical shock stimulus delivered to the left wrist [31]. This thresholding enabled us to create a bespoke shock stimulus for each subject that was mildly painful, but not intolerable, and matched in subjective intensity for all subjects. Immediately after the thresholding, subjects played the role of "decider" in a decision task (Figure 1) where they made 172 decisions involving tradeoffs between profits for themselves and pain for either themselves (Figures 1A and 1C) or an anonymous other "receiver" (Figures 1B and 1D) [12]. We separately manipulated whether participants *increased* pain via a motor action (Figures 1A and 1B) or *decreased* pain via a motor action (Figures 1C and 1D). To avoid habituation and sensitization and preserve choice independence, we delivered no money or shocks during the task. Instead, one trial was selected by the computer and implemented at the end of the experiment. Decisions were completely anonymous and confidential with respect to both the receiver and the experimenters. Subjects were made aware of these details.

Thus, our experimental design allowed us to investigate the drugs' effects on motor impulsivity—i.e., a propensity to respond prematurely before considering the consequences of action [32]—independently from their effects on harm aversion per se. This is important because previous work cannot rule out the possibility that monoamines influence aggression via their evident effects on motor impulsivity [13, 33, 34]. If the link between monoaminergic function and antisocial behavior is mediated by monoaminergic influences on motor impulsivity, then we would expect to see drug effects only in trials where subjects increased harm via action. By contrast, if monoamines influence antisocial behavior through effects on valuation of harmful outcomes, then we would expect to see drug effects in all trials, regardless of action requirements.

Computational Model of Moral Decision Making

We fit a computational model to subjects' choice data and examined the effects of citalopram and levodopa on the model parameters. This approach tests the ability of a hypothesized set of cognitive processes to account for all choices rather than only hand-selected aspects of the data [35]. We used a model independently validated in two previous behavioral studies using the same decision task [12]. The model explained the data well, correctly predicting 84% of deciders' choices in study 1 (95% confidence interval [CI] [83–86]) and 85% of deciders' choices in study 2 (95% CI [84–86]). The model allows for distinct valuation of harms to self and other and incorporates a factor that accounts for loss aversion for both shocks and money:

$$\Delta V = (1-\kappa)\mathscr{L}_m \Delta m - \kappa \mathscr{L}_s \Delta s$$

$$\kappa = \begin{cases} \kappa_{self} \text{ if self trial} \\ \kappa_{other} \text{ if other trial} \end{cases}$$

$$\mathscr{L}_m = \begin{cases} 1 \text{ if } \Delta m > 0 \\ \lambda \text{ if } \Delta m < 0 \end{cases}$$

$$\mathscr{L}_s = \begin{cases} 1 \text{ if } \Delta s < 0 \\ \lambda \text{ if } \Delta s > 0 \end{cases},$$

where ΔV is the subjective value of switching from the default to the alternative option, and Δm and Δs are the objective differences in money and shocks between the default and alternative options, respectively. ΔV is based on a weighted average of these two quantities, where the relative weighting given to Δs is determined by a harm aversion parameter κ. When $\kappa = 0$, deciders will accept any number of shocks to increase profits. As κ approaches 1, deciders become maximally harm averse and will pay increasing amounts to avoid a single shock. The setting of κ depends on who is receiving the shocks, where κ_{self} and κ_{other} capture the subjective cost of pain for self and others, respectively. Finally, the objective differences, Δm and Δs, are modulated by a loss aversion parameter λ that captures the difference in the sensitivity of subjective value to gains (increases in money or decreases in shocks) versus losses (decreases in money or increases in shocks)

CellPress

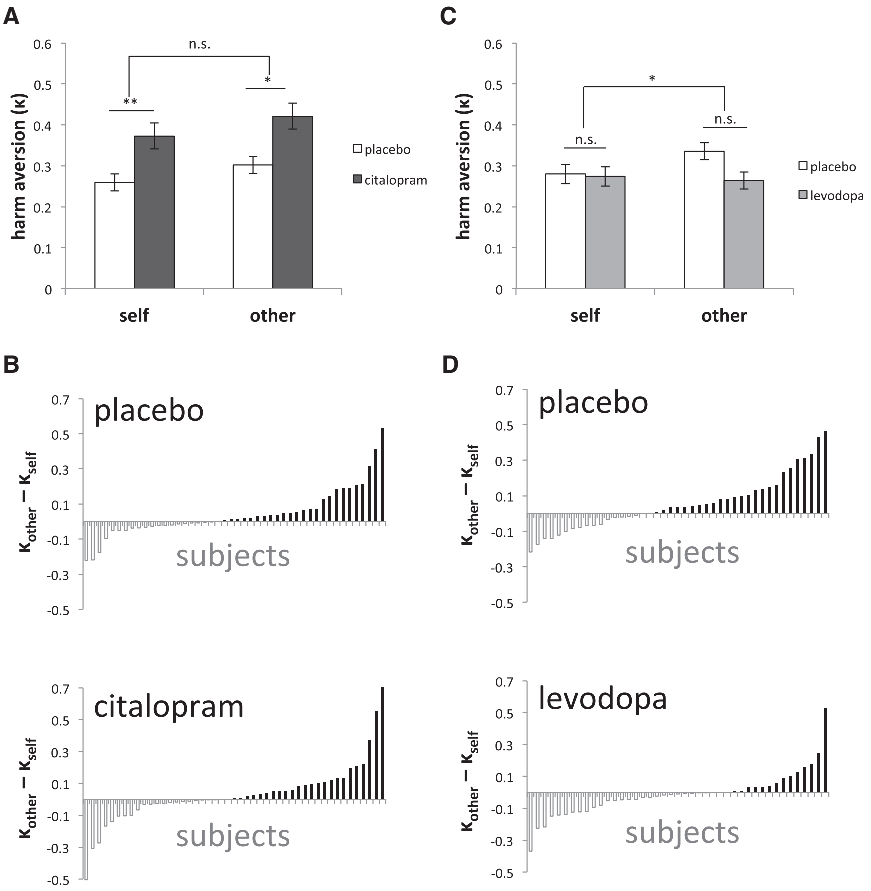

Figure 2. Effects of Citalopram and Levodopa on Harm Aversion and Hyperaltruism in Study 1 and Study 2

(A) In study 1, Citalopram significantly increased harm aversion for self (κ_{self}) and others (κ_{other}) but did not affect the difference in harm aversion between self and others (i.e., hyperaltruism).

(B) Sorted effect sizes of hyperaltruism (defined by taking the difference between κ_{other} and κ_{self}) across participants for the placebo and citalopram groups in study 1. Black bars indicate hyperaltruistic subjects, while white bars indicate selfish subjects.

(C) In study 2, Levodopa did not affect harm aversion for self or others, but significantly decreased the difference in harm aversion between self and others.

(D) Sorted effect sizes of hyperaltruism ($\kappa_{other} - \kappa_{self}$) across participants for the placebo and levodopa groups in study 2.

*$p < 0.05$; **$p < 0.01$; n.s., not significant. Error bars represent SEM difference between κ_{self} and κ_{other}.

[14]. Trial-by-trial value differences were transformed into choice probabilities using a softmax function [36].

Drug Effects on Moral Decisions

We previously observed in this exact setting that subjects were hyperaltruistic, in that harm aversion was greater for others than for self [12]. We replicate this effect here in the placebo groups of both studies (study 1: $t_{(45)} = -2.08$, $p = 0.043$; study 2: $t_{(42)} = -2.37$, $p = 0.023$). Hyperaltruism (computed as the difference in harm aversion for self and others, i.e., $\kappa_{other} - \kappa_{self}$) resulted in subjects being willing to pay, on average, an extra 10p per shock to prevent shocks to others, relative to themselves.

Our primary aim was to examine the effects of citalopram and levodopa on harm aversion for self and others (captured by our model's harm aversion parameters κ_{self} and κ_{other}). We formally tested this in an omnibus mixed-effects ANOVA on the harm aversion parameter estimates with shock recipient (self, other) as a within-subjects factor and study and drug as between-subjects factors. We found a significant dissociation in the effects of citalopram and levodopa on harm aversion for self and others (study × drug interaction, $F_{(1,171)} = 5.268$, $p = 0.023$).

Next, we examined separately the drugs' effects on the harm aversion parameters. Citalopram increased harm aversion both for self and others (main effect of drug, $F_{(1,87)} = 7.114$, $p = 0.009$; κ_{self}, $t_{(87)} = -2.761$, $p = 0.007$; κ_{other}, $t_{(87)} = -2.240$, $p = 0.028$; Figure 2A). There was no interaction between shock recipient and drug ($F_{(1,87)} = 0.016$, $p = 0.90$) indicating that citalopram influenced harm aversion for self and others to the same degree. Correspondingly, subjects on citalopram delivered fewer shocks to themselves and others than those on placebo (main effect of drug, $F_{(1,87)} = 6.220$, $p = 0.015$; shocks to self: $t_{(87)} = -2.673$, $p = 0.009$; shocks to others: $t_{(87)} = -2.353$, $p = 0.021$). The effects of citalopram on harm aversion could not be explained by a reduction in motor impulsivity (Supplemental Information). Strikingly, citalopram nearly doubled the subjective cost per shock, both for self (from 35p to 60p per shock) and others (from 44p to 73p per shock). This resulted in subjects on citalopram choosing to deliver, on average, 30 fewer shocks to themselves and 35 fewer shocks to others over the course of the experiment, relative to subjects on placebo. Levodopa, by contrast, did not significantly affect harm aversion for self or others (κ_{self}, $t_{(42)} = -0.318$, $p = 0.75$; κ_{other}, $t_{(42)} = 1.099$, $p = 0.28$).

We then examined the drugs' effects on hyperaltruism. Planned comparisons indicated that citalopram did not affect hyperaltruism (drug × recipient interaction, $F_{(1,87)} = 0.016$, $p = 0.90$; Figure 2B). By contrast, levodopa reduced hyperaltruism (drug × recipient interaction, $F_{(1,84)} = 4.358$, $p = 0.040$; Figures 2C and 2D), to the extent that subjects in the levodopa group did not show significantly greater harm aversion for others than for self ($t_{(42)} = 0.497$, $p = 0.622$). This resulted in subjects on levodopa choosing to deliver, on average, ten more shocks to the receiver over the course of the experiment, relative to subjects on placebo. Accordingly, subjects tended to deliver fewer shocks to others than themselves on placebo, but not levodopa (drug × recipient interaction, $F_{(1,84)} = 3.048$, $p = 0.084$). The omnibus three-way interaction between study, drug, and shock recipient did not reach significance ($F_{(1,171)} = 2.066$, $p = 0.152$), leaving open the possibility that citalopram may affect hyperaltruism, albeit to a lesser degree than levodopa. The reduction in hyperaltruism

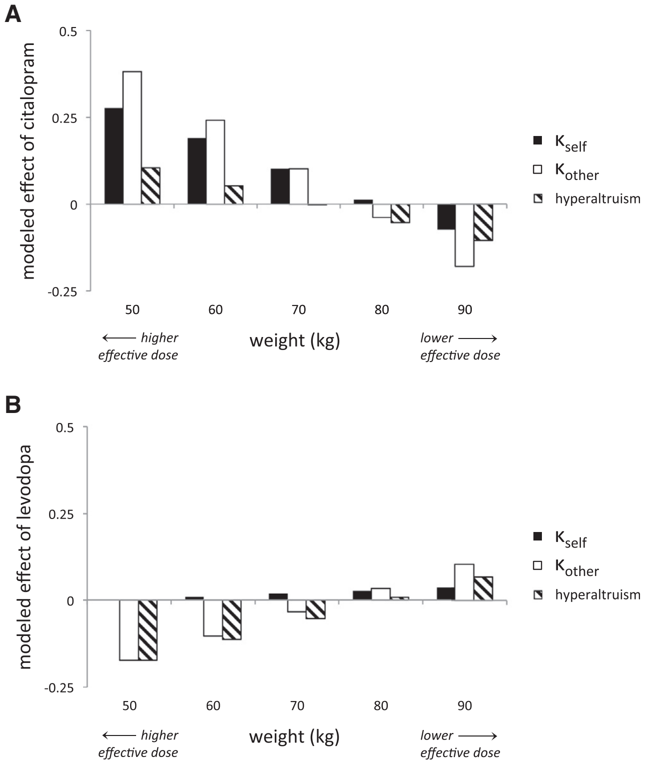

Figure 3. Predictions of Fitted Regression Models of the Interaction of Drug and Effective Dosage on Harm Aversion for Self and Others and Hyperaltruism

(A) Citalopram increased harm aversion for self and others relative to placebo, more strongly for subjects with lower body weight (who thus received a higher effective dose).

(B) Levodopa reduced hyperaltruism relative to placebo, more strongly for subjects with lower body weight (who thus received a higher effective dose).

observed following levodopa could not be explained by increased motor impulsivity (Supplemental Information).

Selectivity of Drug Effects

Importantly, the effects of the drugs on harm aversion could not be attributed to changes in subjective experience of pain, as neither drug affected subjects' pain thresholds (citalopram: $t_{(85)} = -0.4102$, $p = 0.6827$; levodopa: $t_{(84)} = -0.0166$, $p = 0.9868$; Figure S1). Moreover, neither drug affected estimates of the loss aversion parameter λ (citalopram: $z = 1.05$, $p = 0.295$; levodopa: $z = 1.42$, $p = 0.157$).

We performed additional analyses to confirm the selectivity of our effects. Subjective feeling reports on 16 dimensions, measured pre-task and post-task, did not differ significantly for levodopa versus placebo (Table S1). For citalopram versus placebo, we observed increases in the states "feeble," "troubled," and "incompetent," although none survived multiple comparison correction (Table S1). Nevertheless, the effects of citalopram on harm aversion for self and others remained significant when controlling for these state changes (κ_{self}: $\beta = 0.08 \pm 0.04$, $p = 0.047$; κ_{other}: $\beta = 0.11 \pm 0.06$, $p = 0.049$), and none of the mood variables significantly affected harm aversion or interacted with the drug effects (all $p > 0.14$).

Evidence for Dose Dependency of Drug Effects

We also tested for causality in the drugs' effects by examining the influence of effective drug dosage (which varied according to subjects' body weight: 0.31–0.62 mg/kg for citalopram, 1.43–3.35 mg/kg for levodopa). For each drug, we performed a linear regression testing jointly for the effects of drug and the interaction of drug and effective dose, controlling for sex and body mass index, as these factors may themselves be associated with baseline monoaminergic function [37, 38]. For citalopram, this analysis revealed significant effects of drug (κ_{self}, $t_{(81)} = 2.50$, $p = 0.014$; κ_{other}, $t_{(81)} = 3.07$, $p = 0.003$) and drug × effective dose (κ_{self}, $t_{(81)} = -2.03$, $p = 0.046$; κ_{other}, $t_{(81)} = -2.61$, $p = 0.011$), indicating that subjects receiving a larger effective dose showed a greater effect of citalopram on harm aversion for self and others (Table S2; the model's account of this is shown in Figure 3A). There was no effect of sex or sex × drug on harm aversion for self or others (all $p > 0.39$). In a parallel analysis of raw choice data, citalopram reduced the number of shocks delivered to self ($t_{(82)} = 2.20$, $p = 0.031$) and others ($t_{(82)} = 2.65$, $p = 0.01$) and did so as a function of effective dose (self: $t_{(82)} = -1.88$, $p = 0.064$; other: $t_{(82)} = -2.28$, $p = 0.025$).

A similar analysis for levodopa revealed significant effects of drug ($t_{(78)} = -2.22$, $p = 0.030$) and a trend level interaction between drug and effective dose ($t_{(78)} = 1.90$, $p = 0.060$) on hyperaltruism (Table S2; the model's account of this is shown in Figure 3B), suggesting that subjects receiving a larger effective dose showed a greater effect of levodopa on hyperaltruism. There was no effect of sex or sex × drug on hyperaltruism (all $p > 0.45$). A corresponding analysis of raw choice data showed that levodopa significantly reduced the difference in shocks delivered to self versus others ($t_{(79)} = -2.12$, $p = 0.038$) and tended to do so as a function of effective dose ($t_{(79)} = 1.92$, $p = 0.059$).

Drug Effects on Response Times

Neither drug affected overall response times (citalopram: $t_{(87)} = 1.32$, $p = 0.19$; levodopa: $t_{(84)} = -0.254$, $p = 0.80$). Previously we found that hyperaltruism was related to slower decisions for others relative to self [12]. We replicated this finding here (study 1: $r = 0.29$, $p = 0.006$; Figure 4A; study 2: $r = 0.27$, $p = 0.01$; Figure 4B). In light of levodopa's reduction of hyperaltruism, we investigated whether levodopa also reduced slowing for decisions about others. Because the drugs shifted subjects' indifference points in terms of the amount of money they were willing to sacrifice to avoid pain, which resulted in them making different choices under drug versus placebo conditions, we restricted our analysis to trials near subjects' indifference points, examining how the drugs modulated the effects of shock recipient, and differences in subjective value between the choice options, on response times (Table S3). An omnibus ANOVA testing a formal dissociation in the drugs' effects showed a significant interaction between response time component, study, and drug ($F_{(1,171)} = 3.57$, $p = 0.029$), indicating dissociable effects of citalopram and levodopa on response times. Levodopa reduced slowing for others ($t_{(84)} = 2.15$, $p = 0.035$) without affecting speeding related to subjective value differences ($t_{(84)} = -1.09$, $p = 0.278$; Figure 4C). Meanwhile, citalopram reduced speeding related to subjective value differences ($t_{(87)} = -2.23$, $p = 0.028$) without affecting slowing for others ($t_{(87)} = -0.698$, $p = 0.487$; Figure 4D). A separate

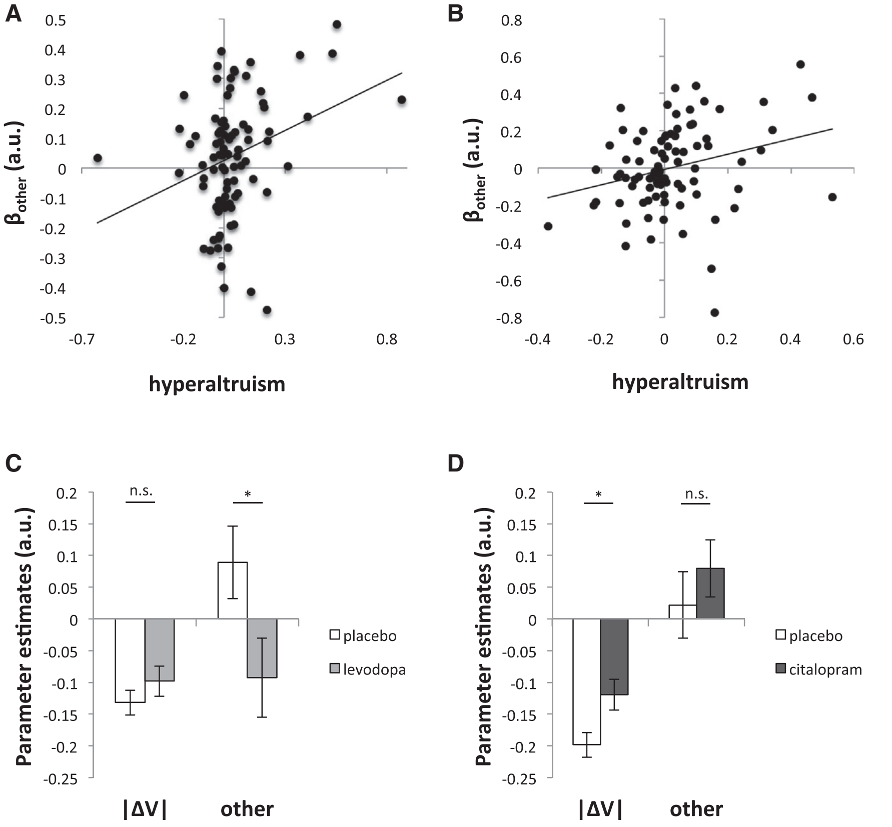

Figure 4. Effects of Hyperaltruism and Drugs on Response Times

(A and B) Hyperaltruism ($\kappa_{other} - \kappa_{self}$) was positively correlated with slower decisions for others relative to self in study 1 (A) and study 2 (B), as measured by the weight of a general linear model regressor indicating whether the outcome was for other (β_{other}).

(C) Levodopa reduced slowing associated with deciding for others, relative to self, but did not affect speeding related to value differences ($|\Delta V|$).

(D) Citalopram reduced speeding related to value differences but did not affect slowing associated with deciding for others, relative to self.

*$p < 0.05$. Error bars represent SEM. a.u., arbitrary units. See also Table S3.

analysis including all trials revealed complementary findings (see Supplemental Information).

DISCUSSION

We combined pharmacological tools with a computational model of harm aversion to show that serotonin and dopamine manifest dissociable neuromodulatory effects on moral decision making. Inhibition of central serotonin reuptake, which increases synaptic serotonin, strongly and selectively increased harm aversion for both self and others. By contrast, increasing central dopamine levels reduced the extent to which people placed others' welfare before their own. The drugs also had dissociable effects on response times, and their effects on behavior are not explained by changes in motor impulsivity or subjective mood. The drugs' effects on model parameters were somewhat stronger than their effects on behaviors in aggregate, highlighting the sensitivity of our model-based approach. Overall, our data provide evidence that serotonin and dopamine modulate moral preferences in distinct ways, with ramifications for understanding prosocial behavior and its disruption in psychiatric disorders.

Our data provide the first direct comparison of serotonergic modulation of harm aversion for self and others. Citalopram increased the subjective cost of harm similarly for self and others, suggesting that serotonin influences social behavior through a general effect on integrating aversive and appetitive values rather than a specific effect on social cognition. This explanation also fits with citalopram's effects on response times. Citalopram reduced a speeding associated with incentive motivation, suggesting that the drug induced a more cautious response disposition, an effect consistent with previous findings [27, 28]. Citalopram also increased negative affect, consistent with serotonin's putative role in mood [29], although we did not find evidence that mood mediated the drugs' effects on harm aversion (Supplemental Experimental Procedures). That citalopram had similar effects on harm aversion for self and others is particularly notable given that self-harm is often comorbid with aggressive, antisocial behavior [39] and that people with psychopathy show deficient responses to punishments to self [1]. A goal for future research is delineating the neural circuitry for computing the relative values of harm to self and others and how this circuitry is modulated by serotonin. Reduced harm aversion may be a risk factor for instrumental and reactive aggression that both feature in psychopathy [40], though serotonin is more strongly implicated in the latter [41].

Levodopa reduced hyperaltruism, albeit to a weaker extent than the effect of citalopram on harm aversion. This finding supports a causal link between phasic dopamine hyperactivity and antisocial behavior in humans [10, 11] and is congruent with past work showing levodopa increases selfishness for monetary reward [26]. Our findings are also consistent with a recent report that enhancing tonic dopamine increased inequality aversion [25]. In the current context, increased inequality aversion would reduce prosociality, since hyperaltruism manifests as a preference for inequality in favor of others. We previously suggested that hyperaltruism might be driven by an uncertainty inherent in decisions affecting others [12]. If subjects assume a nonlinear mapping from objective shocks to subjective utility, then uncertainty about the shape of the receiver's utility function could induce a form of "moral risk aversion" where people err on the side of caution to avoid imposing intolerable costs on others. As uncertainty is associated with slower responding, this explanation gels with observations that hyperaltruism is positively correlated with slower deciding for others relative to oneself [12].

The uncertainty hypothesis suggests a mechanism through which levodopa reduces hyperaltruism and slowing when deciding for others. Dopamine may reduce uncertainty about others' utilities by reducing the variability of neural representations of others [42]. Such a mechanism could be implemented by dopaminergic modulation of the medial prefrontal cortex (mPFC), a region that encodes uncertainty about others' intentions [43] and receives dopamine projections [44].

An alternative explanation relates to dopamine's putative role in safety signaling and active avoidance [20]. Trials where the shocks are assigned to the other rather than oneself could be treated as safety signals, evoking a dopaminergic prediction error that is enhanced by levodopa. Increased dopaminergic tone could then stimulate reward-seeking behavior and increase response vigor [45]. This would result in reduced harm aversion and faster responding when deciding for others relative to oneself. While the uncertainty hypothesis predicts that a prefrontal mechanism would mediate dopamine's effects on hyperaltruism, the safety signaling mechanism would likely be mediated through the striatum. Neuroimaging studies could help resolve these competing explanations.

We capitalized on variation in subjects' body weight to examine the potential effects of effective dosage. This analysis suggested that subjects with lower body weight, who thus received a higher effective dose, showed stronger drug effects on moral decision making. An important caveat is that weight may influence baseline monoaminergic function [37, 38]. We attempted to mitigate potential baseline differences by controlling for sex and BMI in our analyses. The observed interactions between drug and body weight could also result from a biphasic dose-response curve that has been observed previously for citalopram and levodopa [46, 47]. Potential dose dependency of our effects could be more optimally addressed in future studies using a within-subjects design with multiple drug doses.

We have shown that some of the most commonly prescribed psychiatric drugs influence moral decisions, raising important questions about the ethics of pharmacological interventions. A single dose of citalopram nearly doubled the amount of money people were willing to pay to avoid harming others, while a single dose of levodopa eliminated a hyperaltruistic tendency to prefer harming oneself over others. However, it is important to stress that these drugs probably have different effects in healthy volunteers compared to patients, and future work could usefully investigate how serotonin and dopamine influence harm aversion in psychiatric disorders with monoaminergic abnormalities. The model-based approach we employ here is a first step in this direction. These methods enabled us to probe the mechanisms driving neuromodulatory effects on choice and also provide an obvious pathway to future work investigating the neural computations supporting prosocial behavior and its impairment in psychiatric disorders.

EXPERIMENTAL PROCEDURES

Participants

We recruited healthy participants aged 18–35 years, excluding individuals with a history of psychiatric disorders, cardiac or endocrine disorder, medication or drug use (other than contraceptive pills), or previous allergic reactions to medications, individuals who may be pregnant or are breast feeding, or individuals with >1 year studying psychology. Participants were instructed to avoid taking caffeine on the day of the study, alcohol and pain medication 24 hr before the study, and recreational drugs 7 days prior to participation.

95 participants (47 male, 48 female; mean age = 22.3 ± 3.85) took part in study 1 (citalopram). Two participants did not complete the study due to side effects, and four participants were excluded for not believing there was a real receiver or for not finding the shocks aversive. The final analysis included 89 participants (drug n = 43, placebo n = 46). 92 participants (46 male, 46 female; mean age = 22.3 ± 3.53) took part in study 2 (levodopa). Six participants were excluded for not believing there was a real receiver or for not finding the shocks aversive. The final analysis included 86 participants (drug n = 43, placebo n = 43). In both studies, the drug and placebo groups did not differ significantly in terms of sex, age, education, or social and emotional traits (Table S4).

Procedure

The two studies were run in parallel at the Wellcome Trust Centre for Neuroimaging in London and were approved by the University College London Research Ethics Committee (4418/003). Participants completed online questionnaires 1 week before the testing session. Two individuals participated in each session. They arrived at staggered times and were led to separate testing rooms without seeing one another.

Upon arrival, participants gave their written informed consent and underwent a medical exam. Participants were randomly assigned to receive either drug or placebo under double-blind conditions. Subjects were unable to distinguish whether they received drug or placebo (citalopram: χ^2 = 0.36, p = 0.55; levodopa: χ^2 = 0, p = 1.0). Subjective state questionnaires were collected at baseline and at three other times during the study. In study 1, participants received either citalopram (30 mg drops, dissolved in orange juice) or placebo (orange juice). In study 2, participants received either levodopa (187.7 mg "Madopar," comprised of 150 mg levodopa and 37.5 mg benserazide, dissolved in orange juice) or a placebo (vitamin C tablet containing ascorbic acid, lactose, and sucrose, dissolved in orange juice).

The start of the harm aversion task was delayed to coincide with peak drug absorption (3 hr or 60 min after drug administration for citalopram and levodopa, respectively). During the waiting period, participants completed additional questionnaires. Before the task, participants completed a role randomization procedure (described in detail elsewhere [12]) that ostensibly assigned them and the other participant to different roles. Next, they completed a pain thresholding procedure [31] followed by the harm aversion task [12] (Supplemental Experimental Procedures). Next, participants completed a learning task (data not shown). After this, the outcome of one trial was delivered. Before departing, participants completed a debriefing questionnaire.

Data Analysis

We used a model derived from previous studies using the harm aversion task [12] that explained choices in terms of the value difference (ΔV) between the default and alternative options. Trial-by-trial value differences were transformed into choice probabilities using a softmax function [36]:

$$P\,(choose\ alternative) = \left(\frac{1}{1+e^{-\gamma\Delta V}}\right)(1-2\varepsilon)+\varepsilon,$$

where γ is a subject-specific inverse temperature parameter that characterizes the sensitivity of choices to ΔV, and ε is an irreducible noise parameter that captures choice noisiness resulting from factors independent of ΔV (such as inattention). We optimized subject-specific parameters across trials using nonlinear optimization implemented in MATLAB (MathWorks) for maximum likelihood estimation. Parameters were estimated individually for each subject, and summary statistics were calculated from these parameter estimates at the group level, treating each parameter estimate as a random effect [48]. We tested the effects of the drugs on model parameters using t tests (for normally distributed parameters) and nonparametric Wilcoxon rank-sum tests (for non-normally distributed parameters).

Response times were log transformed and Z scored prior to analysis. We modeled response times using a general linear model validated in previous studies using the harm aversion task [12]. We analyzed the first 88 trials in the task, as these trials were identical for all participants. The model contained regressors indicating whether the outcome was for self or other (*other*); the difference in shocks between the default and alternative options (Δs); the difference in money between the default and alternative options (Δm); the maximum number

of shocks that could be delivered (s_{max}); the interaction between the difference in money and difference in shocks between the default and alternative options ($\Delta s \times \Delta m$); the interaction between the difference in shocks between the default and alternative options and whether the outcome was for self or other ($\Delta s \times$ *other*); and a constant. Summary statistics were calculated from regressor beta weights at the group level, treating each beta weight as a random effect [48]. We tested the effects of the drugs on beta weights using t tests.

We performed a separate analysis restricted to trials near subjects' indifference points (i.e., for trials whose shock and money amounts created an indifference point that was within ±0.2 units of the subject's own indifference point). We modeled response times using a general linear model containing regressors indicating whether the outcome was for self or other (*other*); the unsigned difference in subjective value between the two choice options, which was constructed individually for each subject using their model parameters ($|\Delta V|$); and a constant term. We tested the effects of the drugs on beta weights using t tests.

SUPPLEMENTAL INFORMATION

Supplemental Information includes Supplemental Results, Supplemental Experimental Procedures, one figure, and four tables and can be found with this article online at http://dx.doi.org/10.1016/j.cub.2015.05.021.

AUTHOR CONTRIBUTIONS

M.J.C., J.Z.S., Z.K.-N., P.D., and R.J.D. designed the research. J.Z.S., G.S., O.T.O., J.M.G.-R., and C.F. performed the research. M.J.C., J.Z.S., Z.K.-N., J.M.G.-R., and C.F. analyzed the data. All authors contributed to writing the paper.

ACKNOWLEDGMENTS

This work was supported by a Sir Henry Wellcome Postdoctoral Fellowship awarded to M.J.C. (092217/Z/10/Z). Z.K.-N. is supported by a Joint Initiative on Computational Psychiatry and Ageing Research between the Max Planck Society and University College London. P.D. is funded by the Gatsby Charitable Foundation. R.J.D. is supported by the Wellcome Trust (078865/Z/05/Z). The Wellcome Trust Centre for Neuroimaging is supported by core funding from the Wellcome Trust (091593/Z/10/Z).

Received: April 5, 2015
Revised: May 9, 2015
Accepted: May 12, 2015
Published: July 2, 2015

REFERENCES

1. Blair, R.J.R. (2013). The neurobiology of psychopathic traits in youths. Nat. Rev. Neurosci. *14*, 786–799.
2. Gray, K., Waytz, A., and Young, L. (2012). The moral dyad: a fundamental template unifying moral judgment. Psychol. Inq. *23*, 206–215.
3. FeldmanHall, O., Mobbs, D., Evans, D., Hiscox, L., Navrady, L., and Dalgleish, T. (2012). What we say and what we do: the relationship between real and hypothetical moral choices. Cognition *123*, 434–441.
4. Cushman, F., Gray, K., Gaffey, A., and Mendes, W.B. (2012). Simulating murder: the aversion to harmful action. Emotion *12*, 2–7.
5. Cima, M., and Raine, A. (2009). Distinct characteristics of psychopathy relate to different subtypes of aggression. Pers. Individ. Dif. *47*, 835–840.
6. van Erp, A.M.M., and Miczek, K.A. (2000). Aggressive behavior, increased accumbal dopamine, and decreased cortical serotonin in rats. J. Neurosci. *20*, 9320–9325.
7. Brown, G.L., Goodwin, F.K., Ballenger, J.C., Goyer, P.F., and Major, L.F. (1979). Aggression in humans correlates with cerebrospinal fluid amine metabolites. Psychiatry Res. *1*, 131–139.
8. Parsey, R.V., Oquendo, M.A., Simpson, N.R., Ogden, R.T., Van Heertum, R., Arango, V., and Mann, J.J. (2002). Effects of sex, age, and aggressive traits in man on brain serotonin 5-HT1A receptor binding potential measured by PET using [C-11]WAY-100635. Brain Res. *954*, 173–182.
9. Lewis, M.H., Gariépy, J.-L., Gendreau, P., Nichols, D.E., and Mailman, R.B. (1994). Social reactivity and D1 dopamine receptors: studies in mice selectively bred for high and low levels of aggression. Neuropsychopharmacology *10*, 115–122.
10. Alia-Klein, N., Goldstein, R.Z., Kriplani, A., Logan, J., Tomasi, D., Williams, B., Telang, F., Shumay, E., Biegon, A., Craig, I.W., et al. (2008). Brain monoamine oxidase A activity predicts trait aggression. J. Neurosci. *28*, 5099–5104.
11. Buckholtz, J.W., Treadway, M.T., Cowan, R.L., Woodward, N.D., Benning, S.D., Li, R., Ansari, M.S., Baldwin, R.M., Schwartzman, A.N., Shelby, E.S., et al. (2010). Mesolimbic dopamine reward system hypersensitivity in individuals with psychopathic traits. Nat. Neurosci. *13*, 419–421.
12. Crockett, M.J., Kurth-Nelson, Z., Siegel, J.Z., Dayan, P., and Dolan, R.J. (2014). Harm to others outweighs harm to self in moral decision making. Proc. Natl. Acad. Sci. USA *111*, 17320–17325.
13. Dalley, J.W., and Roiser, J.P. (2012). Dopamine, serotonin and impulsivity. Neuroscience *215*, 42–58.
14. Kahneman, D., and Tversky, A. (1979). Prospect theory: an analysis of decision under risk. Econometrica *47*, 263–292.
15. Zaki, J., and Ochsner, K.N. (2012). The neuroscience of empathy: progress, pitfalls and promise. Nat. Neurosci. *15*, 675–680.
16. Hein, G., Silani, G., Preuschoff, K., Batson, C.D., and Singer, T. (2010). Neural responses to ingroup and outgroup members' suffering predict individual differences in costly helping. Neuron *68*, 149–160.
17. Soloff, P.H., Meltzer, C.C., Greer, P.J., Constantine, D., and Kelly, T.M. (2000). A fenfluramine-activated FDG-PET study of borderline personality disorder. Biol. Psychiatry *47*, 540–547.
18. Seo, D., Patrick, C.J., and Kennealy, P.J. (2008). Role of serotonin and dopamine system interactions in the neurobiology of impulsive aggression and its comorbidity with other clinical disorders. Aggress. Violent. Behav. *13*, 383–395.
19. Soderstrom, H., Blennow, K., Sjodin, A.-K., and Forsman, A. (2003). New evidence for an association between the CSF HVA:5-HIAA ratio and psychopathic traits. J. Neurol. Neurosurg. Psychiatry *74*, 918–921.
20. Boureau, Y.-L., and Dayan, P. (2011). Opponency revisited: competition and cooperation between dopamine and serotonin. Neuropsychopharmacology *36*, 74–97.
21. Cools, R., Nakamura, K., and Daw, N.D. (2011). Serotonin and dopamine: unifying affective, activational, and decision functions. Neuropsychopharmacology *36*, 98–113.
22. Plenge, P., Mellerup, E.T., and Laursen, H. (1990). Regional distribution of the serotonin transport complex in human brain, identified with 3H-paroxetine, 3H-citalopram and 3H-imipramine. Prog. Neuropsychopharmacol. Biol. Psychiatry *14*, 61–72.
23. FeldmanHall, O., Dalgleish, T., Thompson, R., Evans, D., Schweizer, S., and Mobbs, D. (2012). Differential neural circuitry and self-interest in real vs hypothetical moral decisions. Soc. Cogn. Affect. Neurosci. *7*, 743–751.
24. Crockett, M.J., Clark, L., Hauser, M.D., and Robbins, T.W. (2010). Serotonin selectively influences moral judgment and behavior through effects on harm aversion. Proc. Natl. Acad. Sci. USA *107*, 17433–17438.
25. Sáez, I., Zhu, L., Set, E., Kayser, A., and Hsu, M. (2015). Dopamine modulates egalitarian behavior in humans. Curr. Biol. *25*, 912–919.
26. Pedroni, A., Eisenegger, C., Hartmann, M.N., Fischbacher, U., and Knoch, D. (2014). Dopaminergic stimulation increases selfish behavior in the absence of punishment threat. Psychopharmacology (Berl.) *231*, 135–141.
27. Crockett, M.J., Clark, L., Apergis-Schoute, A.M., Morein-Zamir, S., and Robbins, T.W. (2012). Serotonin modulates the effects of Pavlovian aversive predictions on response vigor. Neuropsychopharmacology *37*, 2244–2252.
28. Geurts, D.E.M., Huys, Q.J.M., den Ouden, H.E.M., and Cools, R. (2013). Serotonin and aversive Pavlovian control of instrumental behavior in humans. J. Neurosci. *33*, 18932–18939.

29. Dayan, P., and Huys, Q.J.M. (2009). Serotonin in affective control. Annu. Rev. Neurosci. *32*, 95–126.

30. Cooper, J.R., Bloom, F.E., and Roth, R.H. (1974). The Biochemical Basis of Neuropharmacology, Second Edition. (Oxford: Oxford University Press).

31. Vlaev, I., Seymour, B., Dolan, R.J., and Chater, N. (2009). The price of pain and the value of suffering. Psychol. Sci. *20*, 309–317.

32. Evenden, J.L. (1999). Varieties of impulsivity. Psychopharmacology (Berl.) *146*, 348–361.

33. Fonseca, M.S., Murakami, M., and Mainen, Z.F. Activation of dorsal raphe serotonergic neurons promotes waiting but is not reinforcing. Curr. Biol. *25*, 306–315.

34. Robbins, T.W., and Crockett, M.J. (2010). Role of central serotonin in impulsivity and compulsivity: comparative studies in experimental animals and humans. In Handbook of the Behavioral Neurobiology of Serotonin (Handbook of Behavioral Neuroscience), C.P. Müller, and B.L. Jacobs, eds. (Elsevier), pp. 415–427.

35. Huys, Q.J.M., Lally, N., Faulkner, P., Eshel, N., Seifritz, E., Gershman, S.J., Dayan, P., and Roiser, J.P. (2015). Interplay of approximate planning strategies. Proc. Natl. Acad. Sci. USA *112*, 3098–3103.

36. Daw, N.D. (2011). Trial-by-trial data analysis using computational models. In Decision Making, Affect, and Learning: Attention and Performance XXIII, M.R. Delgado, E.A. Phelps, and T.W. Robbins, eds. (Oxford University Press).

37. Wang, G.J., Volkow, N.D., Logan, J., Pappas, N.R., Wong, C.T., Zhu, W., Netusil, N., and Fowler, J.S. (2001). Brain dopamine and obesity. Lancet *357*, 354–357.

38. Hesse, S., van de Giessen, E., Zientek, F., Petroff, D., Winter, K., Dickson, J.C., Tossici-Bolt, L., Sera, T., Asenbaum, S., Darcourt, J., et al. (2014). Association of central serotonin transporter availability and body mass index in healthy Europeans. Eur. Neuropsychopharmacol. *24*, 1240–1247.

39. Jenkins, A.L., McCloskey, M.S., Kulper, D., Berman, M.E., and Coccaro, E.F. (2015). Self-harm behavior among individuals with intermittent explosive disorder and personality disorders. J. Psychiatr. Res. *60*, 125–131.

40. Blair, R.J.R. (1995). A cognitive developmental approach to mortality: investigating the psychopath. Cognition *57*, 1–29.

41. Higley, J.D., Mehlman, P.T., Poland, R.E., Taub, D.M., Vickers, J., Suomi, S.J., and Linnoila, M. (1996). CSF testosterone and 5-HIAA correlate with different types of aggressive behaviors. Biol. Psychiatry *40*, 1067–1082.

42. Friston, K., Schwartenbeck, P., FitzGerald, T., Moutoussis, M., Behrens, T., and Dolan, R.J. (2014). The anatomy of choice: dopamine and decision-making. Philos. Trans. R. Soc. Lond. B Biol. Sci. *369*, 20130481.

43. Yoshida, W., Seymour, B., Friston, K.J., and Dolan, R.J. (2010). Neural mechanisms of belief inference during cooperative games. J. Neurosci. *30*, 10744–10751.

44. Williams, S.M., and Goldman-Rakic, P.S. (1998). Widespread origin of the primate mesofrontal dopamine system. Cereb. Cortex *8*, 321–345.

45. Beierholm, U., Guitart-Masip, M., Economides, M., Chowdhury, R., Düzel, E., Dolan, R., and Dayan, P. (2013). Dopamine modulates reward-related vigor. Neuropsychopharmacology *38*, 1495–1503.

46. Sánchez, C., and Meier, E. (1997). Behavioral profiles of SSRIs in animal models of depression, anxiety and aggression. Are they all alike? Psychopharmacology (Berl.) *129*, 197–205.

47. Contin, M., Riva, R., Martinelli, P., and Baruzzi, A. (1992). Kinetic-dynamic relationship of oral levodopa: possible biphasic response after sequential doses in Parkinson's disease. Mov. Disord. *7*, 244–248.

48. Holmes, A., and Friston, K. (1998). Generalisability, random effects & population inference. Neuroimage *7*, S754.

Current Biology
Supplemental Information

Dissociable Effects of Serotonin and Dopamine on the Valuation of Harm in Moral Decision Making

Molly J. Crockett, Jenifer Z. Siegel, Zeb Kurth-Nelson, Olga T. Ousdal, Giles Story, Carolyn Frieband, Johanna M. Grosse-Rueskamp, Peter Dayan, and Raymond J. Dolan

Supplemental Data

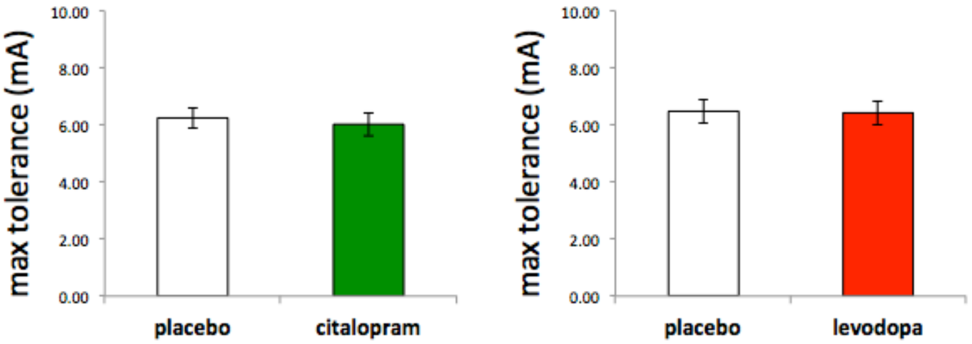

Figure S1, related to Figure 1. Subjects' pain thresholds (the level of stimulation, in mA, corresponding to a 10/10 "intolerable" rating on a subjective pain scale) did not differ between the drug and placebo groups in study 1 (left panel) or study 2 (right panel). Error bars represent SEM. mA, milliamps.

Subjective state questionnaire	Placebo change, study 1	Citalopram change, study 1	P-value, study 1	Placebo change, study 2	Levodopa change, study 2	P-value, study 2
Alert 0 – drowsy 10	-1.51	-0.92	0.23	-0.62	-0.96	0.49
Calm 0 – excited 10	1.15	0.89	0.61	1.44	1.16	0.60
Strong 0 – feeble 10	-0.65	0.40	0.01	0.03	-0.23	0.49
Muzzy 0 – clear headed 10	0.39	0.20	0.70	0.34	-0.33	0.17
Coordinated 0 – clumsy 10	-0.40	-0.42	0.95	-0.15	0.31	0.22
Lethargic 0 – energetic 10	1.15	0.54	0.19	0.96	0.35	0.24
Contented 0 – discontented 10	0.13	0.63	0.20	0.64	0.60	0.88
Troubled 0 – tranquil 10	-0.31	-1.67	0.01	-0.35	-0.69	0.40
Slow 0 – quick witted 10	0.67	0.20	0.21	0.52	0.00	0.28
Tense 0 – relaxed 10	-1.79	-1.58	0.70	-1.35	-1.19	0.73
Attentive 0 – dreamy 10	-1.29	-0.59	0.12	-0.66	-0.24	0.29
Incompetent 0 – proficient 10	0.28	-0.51	0.04	0.36	-0.11	0.29
Happy 0 – sad 10	-0.30	0.32	0.11	0.23	0.36	0.65
Antagonistic 0 – friendly 10	-0.10	-0.75	0.06	-0.39	-0.79	0.41
Interested 0 – bored 10	-0.17	-0.22	0.86	0.28	0.10	0.53
Withdrawn 0 – sociable 10	0.09	0.00	0.79	0.36	0.00	0.46

Table S1, related to Figure 2. Scores represent differences in ratings in a subjective state questionnaire between baseline (before placebo or drug administration) and the start of the task. Questions were answered by marking a point on a line and responses were converted to a 0-10 scale. P-values are not corrected for multiple comparisons. Three variables showed differences (p

< 0.05) between placebo and citalopram (citalopram made subjects feel more feeble, troubled, and incompetent, relative to placebo). None of the observed differences survived correction for multiple comparisons (Bonferroni corrected p = 0.003). None of the variables showed significant differences between placebo and levodopa.

Study 1 (citalopram)						
Regressor	κ_{self}	sh_{self}	κ_{other}	sh_{other}	hyperaltruism	$sh_{self\text{-}oth}$
drug	0.70**	374.06**	1.07***	541.30***	-0.37	-167.24
	(0.28)	(170.29)	(0.35)	(204.56)	(0.26)	(137.02)
male	-0.01	-0.36	-0.08	11.86	-0.07	-47.98
	(0.08)	(50.54)	(0.10)	(60.71)	(0.08)	(40.67)
weight	0.002	3.47	0.006	4.58	-0.002	-1.11
	(0.005)	(2.95)	(0.006)	(3.56)	(0.004)	(2.38)
BMI	-0.0003	1.18	-0.007	2.87	-0.01	-1.69
	(0.01)	(8.19)	(0.02)	(9.85)	(0.01)	(6.60)
drug * male	0.04	67.67	0.04	57.76	0.001	9.91
	(0.10)	(59.20)	(0.12)	(71.12)	(0.09)	(47.63)
drug* weight	-0.01**	-4.96*	-0.01**	-7.23**	0.01	2.27
	(0.004)	(2.64)	(0.005)	(3.17)	(0.004)	(2.12)
constant	0.15	93.82	0.11	-71.10	0.34	164.92
	(0.20)	(136.31)	(0.25)	(163.74)	(0.20)	(109.68)
Study 2 (levodopa)						
Regressor	κ_{self}	sh_{self}	κ_{other}	sh_{other}	hyperaltruism	$sh_{self\text{-}oth}$
drug	-0.02	8.50	-0.46	261.70	-0.45**	-253.19**
	(0.24)	(161.51)	(0.31)	196.05	(0.20)	(120.70)
male	-0.04	7.54	0.001	-10.92	0.04	18.46
	(0.07)	(46.28)	(0.09)	(56.18)	(0.06)	(34.30)
weight	0.001	0.38	-0.004	2.59	-0.005	-2.22
	(0.003)	(1.98)	(0.004)	(2.41)	(0.003)	(1.47)
BMI	-0.0002	0.22	0.0001	0.48	0.001	-0.26
	(0.003)	(1.72)	(0.003)	(2.09)	(0.01)	(1.28)
drug * male	-0.06	43.15	-0.12	87.32	-0.06	-44.16
	(0.09)	(60.54)	(0.12)	(73.49)	(0.08)	(44.87)
drug* weight	0.001	-0.67	0.01	-4.28	0.006*	3.61*
	(0.004)	(2.54)	(0.005)	(3.08)	(0.003)	(1.88)
constant	0.25	372.76	0.58	207.05	0.32	165.71

Table S2, related to Figure 3. Mean parameter estimates for effects of drug, sex, and effective dose (body weight) on harm aversion for self and others, total shocks delivered to self and others,

hyperaltruism, and the difference in total shocks delivered to self vs. others; SEMs in parentheses. ***p < 0.01, **p < 0.05, *p < 0.10

Regressor	Study 1			Study 2		
	**citalopram**	**placebo**	**p-value**	**levodopa**	**placebo**	**p-value**
$\lvert \Delta V \rvert$	-0.12	-0.19	0.03	-0.10	-0.13	0.28
	(0.02)	(0.02)		(0.02)	(0.02)	
other	0.08	0.03	0.49	-0.09	0.09	0.03
	(0.04)	(0.05)		0.06	(0.06)	
constant	-0.34	-0.23	0.18	-0.31	-0.39	0.31
	(0.05)	(0.05)		(0.06)	(0.05)	

Table S3, related to Figure 4. Mean parameter estimates for effects of unsigned value difference ($\lvert \Delta V \rvert$) and shock recipient (*other*) on response times; SEMs in parentheses. P-values indicate the results of unpaired *t*-tests comparing parameter estimates for drug and placebo groups.

Study 1	Placebo (N=46)	Citalopram (N=43)	p-value
**Age (SD)**	22.30 (3.140)	22.47 (4.522)	0.845
**Gender %**			0.924
Male	22 (47.8%)	21 (48.8%)	
Female	24 (52.2%)	22 (51.2%)	
**Education (SD)**	4.28 (1.452)	3.84 (1.511)	0.156
**Behavioural Activation Scale (SD)**	41.59 (4.665)	40.44 (4.339)	0.235
**Behavioural Inhibition Scale (SD)**	20.93 (4.030)	22.21 (2.875)	0.091
**Interpersonal Reactivity Index (SD)**	78.61 (10.472)	78.26 (9.878)	0.871
**Altruism Scale (SD)**	60.61 (6.275)	59.95 (5.884)	0.616
**Social Desirability Scale (SD)**	39.59 (7.776)	40.95 (6.222)	0.365
**Psychopathy Scale (SD)**	121.87 (23.210)	114.72 (22.230)	0.142
**Personality Belief Questionnaire (SD)**	143.61 (35.642)	138.95 (29.105)	0.503
**Personality Inventory for DSM-5 (SD)**	49.11 (9.823)	48.35 (8.685)	0.701
**Inventory of Interpersonal Problems (SD)**	69.33 (16.075)	69.09 (14.044)	0.942

Study 2	Placebo (N=43)	Levodopa (N=43)	p-value
**Age (SD)**	22.14 (3.060)	22.56 (3.978)	0.586
**Gender %**			0.388

Male	19 (44.2%)	23 (53.5%)	
Female	24 (55.9%)	20 (46.5%)	
**Education (SD)**	4.40 (1.330)	4.21 (1.283)	0.511
**Behavioural Activation Scale (SD)**	40.86 (4.533)	39.37 (4.986)	0.151
**Behavioural Inhibition Scale (SD)**	21.19 (2.780)	20.21 (3.967)	0.190
**Interpersonal Reactivity Index (SD)**	77.16 (9.978)	74.79 (12.881)	0.343
**Altruism Scale (SD)**	61.28 (4.763)	60.81 (11.289)	0.804
**Social Desirability Scale (SD)**	40.91 (6.328)	40.70 (7.163)	0.886
**Psychopathy Scale (SD)**	126.56 (22.255)	124.19 (29.436)	0.674
**Personality Belief Questionnaire (SD)**	146.05 (34.239)	148.16 (34.259)	0.775
**Personality Inventory for DSM-5 (SD)**	48.95 (10.415)	47.14 (8.983)	0.390
**Inventory of Interpersonal Problems (SD)**	71.49 (17.644)	64.35 (19.303)	0.077

Table S4. Demographic characteristics of drug and placebo groups in studies 1 and 2

Supplemental results: motor impulsivity, related to Figure 2

To examine whether the observed serotonergic modulation of harm aversion could be explained by reduced motor impulsivity, we fit separate harm aversion parameters for self and others on trials where action increased harm and trials where inaction increased harm, and tested the effects of citalopram on harm aversion in a mixed ANOVA with shock recipient (self, other) and action (increase, decrease) as within-subjects factors, and drug (citalopram, placebo) as a between-subjects factor. This analysis ruled out the possibility that citalopram increased harm aversion by reducing impulsivity, as we observed a main effect of citalopram on harm aversion ($F_{(1,87)} = 7.910$, $p = 0.006$) but no interaction between drug and action ($F_{(1,87)} = 0.042$, $p = 0.838$).

The reduction in hyperaltruism observed following levodopa could not be explained by increased motor impulsivity. We fit separate harm aversion parameters for self and others on trials where actions increased and decreased harm and computed hyperaltruism separately for these increasing and decreasing trials, as in the above analysis with citalopram. We observed a main effect of levodopa on hyperaltruism ($F_{(1,84)} = 4.104$, $p = 0.046$) but no interaction between drug and action ($F_{(1,84)} = 1.126$, $p = 0.292$).

Supplemental results: response times, related to Figure 4

We were able to examine whether the drugs moderated the influences of harm, profits, and shock recipient on response times because these factors were varied independently across trials. We describe here the results of an analysis that includes all trials, i.e., not just those around subjects' indifference points. In a general linear model testing the effects of levodopa on changes in response times related to harm, profits, shock recipient, and interactions between these factors, the effect of levodopa on slowing for others was not significant ($t_{(84)} = -1.19$, $p = 0.236$). However, we observed an effect of levodopa on the speed of decisions that specifically involved increasing harm to others. On those trials, levodopa reduced slowing for others relative to self as a function of increasing harm magnitude ($t_{(81)} = -2.09$, $p = 0.042$, corrected for multiple comparisons). Citalopram did not affect the speed of decisions involving increasing harm to others ($t_{(75)} = 0.27$, $p = 0.786$).

The effect of citalopram on response times is consistent with a role for serotonin in aversive processing. Subjects were generally faster when responding resulted in greater profit ($\beta_{\Delta m} = -0.04 \pm 0.002$, $t_{(88)} = -18.12$, $p = 2e^{-31}$). This incentive-induced speeding was reduced when responding also resulted in relatively greater harm (i.e., we observed an interaction between profit and harm on response times; $\beta_{\Delta s*\Delta m} = 0.002 \pm 4e^{-4}$, $t_{(88)} = 3.97$, $p = 0.0001$). This latter effect, whereby the presence of harm reduced incentive motivation, was enhanced by citalopram ($t_{(42)} = -2.70$, $p = 0.008$), in line with previous findings implicating serotonin in behavioral inhibition in the face of aversive expectations, i.e., aversive Pavlovian-to-instrumental transfer [S1, S2].

It is worth noting that the effects of levodopa and citalopram on response times when considering all trials together differed slightly from the drugs' effects on response times when considering only trials around subjects' indifference points. However, these different analytical approaches, which are influenced by ceiling and floor effects in different ways, revealed effects that point in the same direction. In both cases, levodopa reduced components of slowing when deciding for others relative to oneself, whilst citalopram enhanced aspects of behavioral inhibition. Further research is needed to tease apart the finer aspects of how levodopa and citalopram influence the speed of decisions involving harm to self or others.

Supplemental Experimental Procedures

Pain thresholding procedure

Participants underwent individual pain titration procedures with a Digitimer DS5 electric stimulator. Following a brief overview of the equipment and titration process, two electrodes

were placed on the back of the participant's left wrist. Titration began with a low-intensity electric shock (0.1 mA) and subjects were asked to rate their experience of pain on an 11-point scale (ranging from 0 = no pain to 10 = intolerable). The initial rating was followed by a series of shocks, either increasing or decreasing in small milliamp increments with a 3:1 ratio. Subjective ratings of pain were collected after each shock until a rating of 10 was reached, which was recorded as the maximum threshold. Titration was repeated three times for every participant.

Next, we fit a sigmoid function to a series of shocks, allowing us to estimate the current-to-rating response curve. To do this, we generated a series of seven shocks that ranged from 40 to 100 percent of the subject's maximum threshold in 10% increments. Each of the seven shock intensities was delivered three times in random order. From the derived function we estimated the current intensity that corresponded to each participant's level 8 pain experience. The stimulation level corresponding to a subjective level 8 was used to deliver shocks based on the outcomes in the decision-making task. The titration procedure also served to provide an explicit experience of the aversive stimulus, thus allowing subjects to make meaningful judgments and decisions throughout the task.

We note that phasic pain stimulation is widely used in research settings [S3, S4]. We used electric stimulation because it is consistently judged as aversive across participants and remains so throughout the course of an experiment. Furthermore, electric shocks are not common stimuli in daily life; thus, no existing monetary value could be associated with them.

Moral decision task

All participants were instructed that they were assigned to the role of decider and that the other participant in the session was assigned to the role of receiver. In fact all participants played the role of decider. Behavior in the placebo conditions of the current study was no different from that in previous studies using this paradigm that did not use deception [S5]. We excluded participants who did not believe there was another participant present in the lab; the proportion of participants excluded for this reason was no different from that in previous studies that did not use deception [S5].

The task contained a total of 172 trials. Each trial consisted of a choice between a default amount of shocks and money and an alternative amount of shocks and money. The first 88 trials were a fixed set of choices that were presented to all subjects. To create these we first created a set of 22 trials, each containing a pair of choices that matched the indifference point of a specific κ value (where κ is a harm aversion parameter in our computational model of decision-making describing the exchange rate between money and pain). Across a set of κ values evenly

distributed across the range of κ values observed in previous studies [S5] (from 0 to 1), for each κ value we generated 10,000 random pairs of positive shock movements Δs and positive money movements Δm and selected the pair [Δs, Δm] closest to the indifference point of that κ value.

Next, these optimized pairs [Δs, Δm] were transformed into choices containing default amounts of shocks and money (s_d and m_d) and alternative amounts of shocks and money (s_a and m_a) as follows: s_d was a positive integer between 0 and 20, randomly drawn from a uniform discrete distribution with the constraint that $0 < s_d + \Delta s < 20$. Similarly, m_d was a positive number between 0 and 20, randomly drawn from a uniform discrete distribution, rounded to the nearest 10th and constrained such that $0 < m_d + \Delta m < 20$. s_a and m_a were then set by adding Δs and Δm to s_d and m_d, respectively. Following this process we had a set of 22 "increase" trials where $s_a > s_d$ and $m_a > m_d$.

We next created a set of 22 mirror-image "decrease" trials by swapping the values of s_a and s_d, and likewise m_a and m_d. Each trial was then presented twice, once in the "self" condition and once in the "other" condition, for a total of 88 trials.

The second 84 trials contained 44 trials generated in a manner similar to the first 88 trials, interspersed with an additional 40 trials that were individually tailored to each subject. These latter trials were included to provide more precise estimates of subjects' harm aversion parameters (κ_i) across the four experimental conditions (self-increase, self-decrease, other-increase, other-decrease). To create these trials, we fit a computational model (described below) to the first 88 trials. We then used the harm aversion parameter estimates derived from the model to create a set of 10 trials within +/- 0.1 units of subjects' κ_i estimate for each experimental condition. These were created by selecting a κ value within +/- 0.1 units of κi, generating 10,000 random pairs of positive shock movements Δs and positive money movements Δm, and then selecting the pair [Δs, Δm] closest to the indifference point of that κ value. We note that previous studies using variants of this paradigm have shown that decision-making in this task is unaffected by whether subjects receive a fixed set of trials or an individually tailored set of trials [S5]. Furthermore, subjects' parameter estimates in the placebo condition of the current study were similar to those observed in previous studies using variants of this paradigm.

Experimental design

We employed a between-subjects design, whereby each subject took part in a single testing session where they were randomly assigned to receive either drug or placebo before completing the harm aversion task. We note that a more powerful approach to investigate drug effects on behavior is to use a within-subjects design, whereby each subject receives both drug and placebo

across two testing sessions. Within-subjects designs allow for a more straightforward interpretation of how effective dosage and subjective state moderate the drug effects, as each subject's change in parameter estimates between the drug and placebo sessions can be plotted against each subject's body weight and subjective state. Unfortunately, a within-subjects design was not possible for the present study due to features of our task design, which ensures that choices are made within an incentive-compatible framework. This is because if subjects learn in session 1 that they will be trading money for pain, they would have an incentive in the session 2 to report a lower pain threshold than their true threshold, in order to maximize their profits. Because it was essential for our study that choices were incentive-compatible, we were limited to a between-subjects design. As such, we had to resort to modelling the effects of effective dosage and subjective state using linear regressions as reported here.

Subjective state analysis

We collected subjective feeling reports on 16 dimensions before and after the task to measure potential drug effects on subjective mood. We did not have any a priori hypotheses about whether citalopram or levodopa would affect subjective mood, as previous studies using identical doses of these drugs in a similar study population did not find drug effects on subjective mood states [S6–S9]. However, we found that citalopram increased subjective feelings of being troubled, feeble and incompetent (though these effects did not survive correction for multiple comparisons). To investigate the possibility that mood changes induced by citalopram mediated the effects of the drug on harm aversion, we tested whether the drug effects on harm aversion remained significant when controlling for mood changes, and whether mood changes interacted with the effect of drug on harm aversion. Our results do not suggest that mood changes mediated the effects of citalopram on harm aversion, as the effect of citalopram on harm aversion remained significant after controlling for changes in mood, and there were no significant interactions between drug and mood changes.

If anything, the mood changes induced by citalopram could have masked the drug's effect on harm aversion, particularly for others. Many studies have shown that prosocial behavior is related to positive mood [S10–S12], and prosocial behavior is negatively associated with feelings of incompetence and self-efficacy [S13, S14]. Thus, previous work predicts that feelings of being troubled, feeble and incompetent should reduce subjects' willingness to sacrifice money to reduce the pain of others, whereas citalopram increased this behavior. Although an investigation of how subjective feeling states influence harm aversion for self and others was beyond the scope of the current study, this remains an important topic for future research.

Supplemental References

S1. Crockett, M. J., Clark, L., Apergis-Schoute, A. M., Morein-Zamir, S., and Robbins, T. W. (2012). Serotonin modulates the effects of Pavlovian aversive predictions on response vigor. Neuropsychopharmacology *37*, 2244–2252.

S2. Geurts, D. E. M., Huys, Q. J. M., Ouden, H. E. M. den, and Cools, R. (2013). Serotonin and Aversive Pavlovian Control of Instrumental Behavior in Humans. J. Neurosci. *33*, 18932–18939.

S3. Vlaev, I., Seymour, B., Dolan, R. J., and Chater, N. (2009). The price of pain and the value of suffering. Psychol. Sci. *20*, 309–317.

S4. Story, G. W., Vlaev, I., Seymour, B., Winston, J. S., Darzi, A., and Dolan, R. J. (2013). Dread and the Disvalue of Future Pain. PLoS Comput Biol *9*, e1003335.

S5. Crockett, M. J., Kurth-Nelson, Z., Siegel, J. Z., Dayan, P., and Dolan, R. J. (2014). Harm to others outweighs harm to self in moral decision making. Proc. Natl. Acad. Sci. *111*, 17320–17325.

S6. Crockett, M. J., Clark, L., Hauser, M. D., and Robbins, T. W. (2010). From the Cover: Serotonin selectively influences moral judgment and behavior through effects on harm aversion. Proc. Natl. Acad. Sci. *107*, 17433–17438.

S7. Guitart-Masip, M., Chowdhury, R., Sharot, T., Dayan, P., Duzel, E., and Dolan, R. J. (2012). Action controls dopaminergic enhancement of reward representations. Proc. Natl. Acad. Sci. *109*, 7511–7516.

S8. Chamberlain, S. R., Müller, U., Blackwell, A. D., Clark, L., Robbins, T. W., and Sahakian, B. J. (2006). Neurochemical Modulation of Response Inhibition and Probabilistic Learning in Humans. Science *311*, 861–863.

S9. Sharot, T., Guitart-Masip, M., Korn, C. W., Chowdhury, R., and Dolan, R. J. (2012). How Dopamine Enhances an Optimism Bias in Humans. Curr. Biol. *22*, 1477–1481.

S10. Carlson, M., Charlin, V., and Miller, N. (1988). Positive mood and helping behavior: A test of six hypotheses. J. Pers. Soc. Psychol. *55*, 211–229.

S11. Rand, D. G., Kraft-Todd, G., and Gruber, J. (2015). The Collective Benefits of Feeling Good and Letting Go: Positive Emotion and (dis)Inhibition Interact to Predict Cooperative Behavior. PLoS ONE *10*, e0117426.

S12. Isen, A. M., and Levin, P. F. (1972). Effect of feeling good on helping: Cookies and kindness. J. Pers. Soc. Psychol. *21*, 384–388.

S13. Caprara, G. V., Alessandri, G., and Eisenberg, N. (2012). Prosociality: The contribution of traits, values, and self-efficacy beliefs. J. Pers. Soc. Psychol. *102*, 1289–1303.

S14. Caprara, G. V., Alessandri, G., Di Giunta, L., Panerai, L., and Eisenberg, N. (2010). The contribution of agreeableness and self-efficacy beliefs to prosociality. Eur. J. Personal. *24*, 36–55.
